# Dose Reduction of IL17 and IL23 Inhibitors in Psoriasis (BeNeBio study): An International, Pragmatic, Multicentre, Randomised, Controlled, Non-Inferiority Trial

**DOI:** 10.1016/j.lanepe.2026.101721

**Published:** 2026-06-01

**Authors:** Juul M.P.A. van den Reek, Charlotte A.M. van Riel, Rani Soenen, Anke Eylenbosch, Lisa Schots, Lara S. van der Schoot, Lynda Grine, Gerjon Hannink, Fabienne Willaert, Tom Hillary, Paula P.M. van Lümig, Pierre-Dominique Ghislain, Paul M. Ossenkoppele, Sven Lanssens, Bas S.P. Prens, Linda Temmerman, Martijn B.A. van Doorn, Ella A.M. van der Voort, Inge Haeck, Janneke Huizinga, Arjen F. Nikkels, Laurence Dierckxsens, Maartje A.M. Berends, Sharon R.P. Dodemont, Jorn Bovenschen, Jo L.W. Lambert, Elke M.G.J. de Jong

**Affiliations:** a Department of Dermatology, Radboud University Medical Centre, Nijmegen, the Netherlands; b Department of Dermatology, Ghent University Hospital, Ghent, Belgium; c Department of Medical Imaging, Radboud University Medical Centre, Nijmegen, the Netherlands; d Department of Dermatology, Erasme Hospital, Brussels, Belgium; e Department of Dermatology, University Hospital Leuven, Leuven, Belgium; f Department of Dermatology, MUMC+, Maastricht, the Netherlands; g Department of Dermatology, Cliniques Saint-Luc, Brussels, Belgium; h Department of Dermatology, ZGT, Almelo, the Netherlands; i Department of Dermatology, Dermatologie Maldegem, Maldegem, Belgium; j Department of Dermatology, Amphia Hospital, Breda, the Netherlands; k Department of Dermatology, AZ Maria Middelares, Ghent, Belgium; l Department of Dermatology, ErasmusMC, Rotterdam, the Netherlands; m Department of Dermatology, Bravis Hospital, Bergen op Zoom, the Netherlands; n Department of Dermatology, UMCU, Utrecht, the Netherlands; o Department of Dermatology, UMCG, Groningen, the Netherlands; p Department of Dermatology, CHU de Liège, Liège, Belgium; q Department of Dermatology, AZ Sint-Lucas, Ghent, Belgium; r Department of Dermatology, Slingeland Hospital, Doetinchem, the Netherlands; s Department of Dermatology, Catharina Hospital, Eindhoven, the Netherlands; t Department of Dermatology, Máxima MC, Eindhoven, the Netherlands

**Keywords:** Biologics, psoriasis, Dose reduction, dosing, randomised controlled trial

## Abstract

**Background:**

Interleukin (IL)17 and IL23 inhibitors (i) for psoriasis are highly effective but not all patients may need the registered dose. Dose reduction (DR) could contribute to prevent unnecessary drug exposure and lower healthcare expenditures. The aim of this study was to evaluate whether DR through stepwise interval prolongation is effective and safe in patients with psoriasis with stable low disease activity.

**Methods:**

This pragmatic, open-label, controlled, non-inferiority randomised clinical trial was performed in 19 (non-)academic hospitals in the Netherlands and Belgium. Patients were randomised (2:1) to stepwise DR or usual care (UC) by block randomisation with random allocation sequence, stratified by biologic. Patients with stable low disease activity on registered dosages of IL17i (secukinumab, ixekizumab, bimekizumab, brodalumab) or IL23i (guselkumab, risankizumab, tildrakizumab) were eligible. In DR, injection intervals were prolonged stepwise to 67.0% and 50.0% of the original dose if disease activity permitted. The primary aim was to assess non-inferiority of this DR strategy compared to UC regarding the incidence proportion of persistent flares (Psoriasis Area and Severity Index (PASI) > 5 for ≥3 months) after 18 months, with a non-inferiority margin of 15.0%. Patients were analysed by intention-to-treat (ITT) and per-protocol (PP) with imputation of missings. ClinicalTrials.gov Identifier NCT04340076; status: closed.

**Findings:**

Between June 30, 2020 and September 14, 2023, 244 patients were included (mean age 51 ± 15 years; 67.0% male; DR N = 164, UC N = 80). After 18 months, a difference of 2.4% (95% CI −2.0% to 6.8%) in incidence proportion of persistent flares (DR (4.0%) and UC (1.6%) in ITT) showed non-inferiority of DR. No serious adverse events/deaths related to DR were reported.

**Interpretation:**

Disease-activity guided, stepwise interval prolongation of IL17i and IL23i in patients with controlled psoriasis is an effective and safe strategy to reduce unnecessary exposure to these expensive drugs.

**Funding:**

ZonMw (the Netherlands Organization for Health Research and Development), KCE Trials (the Belgian Health Care Knowledge Centre).

**Research in context**

**Evidence before this study**

A literature search was performed in PubMed between January 2020 and May 2026 including a search string with terms on psoriasis, all available biologic therapies, and verbs associated with dose reduction (DR). Studies providing full text original research data on DR of biologics in adults with psoriasis who were initially treated according to the registered dose, were included. DR was defined as administration of a lower dose per administration or injection interval prolongation. DR of TNFα inhibitors (i) (adalimumab, etanercept) and the interleukin (IL) 12/23 inhibitor (i) (ustekinumab) by injection interval prolongation was extensively investigated and was found (cost-)effective and safe. Evidence on DR of IL17i and IL23i was limited to case reports or smaller cohorts on DR of IL17i, and to a lesser extent of IL23i. There was one randomised controlled trial on guselkumab in which superresponders were randomised to interval doubling versus normal dose. The evidence on DR of IL17i/IL23i showed promising results regarding effectiveness and safety. The scientific gap in evidence on biologic DR remains the absence of randomised trials providing scientific evidence on a clear stepwise DR strategy for all IL17i and IL23i.

**Added value of this study**

This study is the first randomised, controlled, pragmatic trial designed to investigate non-inferiority of DR of all registered IL17i and IL23i compared to usual care in patients with psoriasis. DR was performed by stepwise prolongation of the injection interval guided by disease activity and quality of life. DR of IL17i and IL23i was found to be non-inferior to usual care in adults with psoriasis who had low or absent disease activity and a good quality of life, without serious adverse events related to DR. The pragmatic character of the trial and the extensive measurements, including serum samples, during the 3-monthly study visits over 18 months, enabled assessments on effectiveness and safety of the dose reduction strategy under circumstances close to usual care. In addition, this research will enable cost-effectiveness analyses, and development of pharmacokinetic and -dynamic models for the newest biologics used in the treatment of psoriasis.

**Implications of all the available evidence**

This randomised prospective study gives the first high-quality, scientific evidence that DR of IL17i and IL23i is effective and safe in daily clinical practice for well-controlled psoriasis patients. The encouraging results support further implementation of biologic DR in real-world clinical practice and highlight the potential for personalised treatment approaches in psoriasis management.

## Introduction

Biologics expanded the treatment armamentarium for patients with moderate-to-severe psoriasis, an immune-mediated inflammatory disease involving skin, nails, and joints. They significantly reduced patients’ disease activity, and improved their quality of life. The newer generation of biologics (i.e. interleukin (IL) 17 inhibitors (i), and IL23i) are highly effective, however, chronic use of these expensive biologics might contribute to unnecessary drug exposure placing a high financial burden on health care systems. Therefore, adequate use of these drugs is warranted, posing the question if stepwise disease-guided dose reduction (DR) is a viable and practical strategy for patients with moderate-to-severe psoriasis.

Previous studies have shown that DR by stepwise prolongation of the injection interval of adalimumab, etanercept, and ustekinumab in patients with psoriasis with stable low disease activity was (cost-)effective, and safe.^1^^,^^2^ However, studies on DR of IL17i and IL23i are scarce, and most of these studies were not randomised, were limited to one biologic, and/or lacked a clear DR strategy.^3^, ^4^, ^5^, ^6^, ^7^, ^8^ Despite the scarcity of evidence on DR of IL17i and IL23i, it has already been applied in clinical practice but without a clear strategy or guideline, resulting in a trial-and-error process.^9^^,^^10^

Our hypothesis was that IL17i and IL23i injection intervals could be effectively and safely prolonged in a substantial part of patients with stable psoriasis by tightly monitoring disease activity, and dermatological quality of life. The primary aim of this randomised trial was to investigate if DR of IL17i and IL23i by a disease activity-guided strategy is non-inferior to usual care (UC) regarding persistent disease flares in patients with psoriasis with low disease activity.

## Methods

### Study design

We conducted a, multicentre, pragmatic, open-label, randomised, controlled, non-inferiority trial of IL17 inhibitors (IL17i) (secukinumab, ixekizumab, brodalumab, bimekizumab) and IL23 inhibitors (IL23i) (guselkumab, risankizumab, tildrakizumab) from June 30, 2020, to January 30, 2025, in 11 Dutch and 8 Belgian (non-)academic hospitals. Patient representatives were involved in all phases of the study. An extended description of the study design, including a rationale for the sample size calculation and non-inferiority margin, is published previously elsewhere (online study protocol).^11^

The study protocol and amendments were approved by the Medical Ethical Committee (Arnhem-Nijmegen, NL71920.091.19) for the Dutch sites and from the competent authorities (FAHMP) and the Ethics Committee of Ghent University Hospital and Ghent University after consulting the Ethics Committees of each Belgian participating site. Study performance was in accordance with Good Clinical Practice guidelines, and the Declaration of Helsinki.^12^ Reporting of this trial is according to the Consolidated Standards of Reporting Trials (CONSORT) reporting guideline. The study is closed and registered in ClinicalTrials.gov (Identifier: NCT04340076; status: closed).

### Patients

Adult patients with plaque psoriasis using standard, registered doses of IL17i or IL23i for at least 6 months with stable low disease activity (defined as Psoriasis Area and Severity Index (PASI) ≤5 for ≥6 months at inclusion, and Dermatology Life Quality Index (DLQI) ≤5 at inclusion) were included. At regular visits, patients were first asked by their treating physicians if they agreed to be informed on the study. If so, patients were further counselled and eventually recruited by trained research staff (research nurses and physicians), who were also actively involved during study follow-up visits. Patients were excluded if the biologic was primarily prescribed for another disease than plaque psoriasis (e.g. for psoriatic arthritis), if they used concomitant systemic immunosuppressants/therapies (except methotrexate and acitretin when used for psoriasis), or if they had a severe comorbidity with short life expectancy. Sex/gender data were derived from patient records; ethnicity/race data were not collected. All patients provided oral and written informed consent.

### Randomisation and masking

A total of 244 patients was randomised 2:1 by a web-based program (www.castoredc.com) to DR (N = 164) or UC (N = 80) by block randomisation (variable block size of 6, 9, 12) with a random allocation sequence, and stratified by biologic. During the entire study, no masking was applied owing to the pragmatic study design aiming for the highest possible external validity. In this way, we aimed to provide a more realistic reflection of how our DR intervention would function in real-world practice rather than under ideal circumstances. Trained physicians or nurses enrolled patients and ensured randomisation by the web-based program, and to ensure internal consistency, they executed the patients’ study follow-up visits.

### Procedures

The DR strategy and study measures are visualised in Supplementary Figure S1 (Supplementary Figure S1). Patients in the UC group received the standard dose of their IL17i or IL23i. Due to the pragmatic design of this study, treatment alterations were allowed when needed as UC represented daily practice care as described previously.^11^ In the DR group, DR was performed by stepwise injection interval prolongation to first 67.0% of the standard dose (1.5-fold extension of the interval in standard dose) (Supplementary Figure S1). Disease activity was monitored at every study visit using PASI and DLQI scores, assessed by trained physicians or nurses. The dose was further reduced to 50.0% of the standard dose (2-fold extension of the interval in standard dose) when disease activity remained low (PASI ≤5 and DLQI ≤5) (Supplementary Figure S1). When PASI >5 and/or DLQI >10, or by patient preference, the (previous) effective dose was reinstalled, meaning reescalation of the dose. When PASI ≤5 and DLQI >5 but ≤10, it was discussed with the patient if the lowered dose could be continued. After DR failure, and consequently, reescalation, no new DR attempts were advised by the study team. Study visits were three-monthly during a total study follow-up of 18 months for both groups. Additional study visits took place when needed, patients were informed on the possibility to contact the study team in-between visits prior to inclusion. At every study visit, questionnaires were completed, and serum samples collected. In both groups, it was permitted to initiate, continue, or discontinue methotrexate, acitretin, or topical therapies for psoriasis when deemed necessary by the physician.

### Outcomes

The primary outcome was to assess non-inferiority of DR by the difference in incidence proportion of persistent flares (PASI >5 for ≥3 consecutive months) between the DR and UC group after 18 months. This difference was compared with the non-inferiority margin of 15.0%. If the upper limit of the 95% confidence interval (CI) of the between-group difference exceeded 15.0%, then non-inferiority of DR was not demonstrated. The primary estimand reflects a treatment-policy strategy, estimating the effect of initiating the DR strategy versus UC on the incidence of persistent flares regardless of treatment modifications after randomisation in all randomised patients over 18 months.

Secondary outcomes were:

• proportion of patients with successful DR (using a lower dose and maintaining PASI ≤5) after 12 and 18 months,
• between-group difference in PASI scores at each three-monthly time point, and in the course of PASI and DLQI scores over 18 months,
• to assess the time until first persistent flare (PASI >5 for ≥3 consecutive months),
• between-group difference in the incidence of short disease flares (PASI >5 at one time point) after 18 months,
• between-group difference in the proportion of patients with initiation of other anti-psoriatic medication (methotrexate, acitretin, cyclosporin, intensive topical therapies like dithranol),
• serious adverse events (SAE) related to DR and between-group differences in SAE, adverse events (AE), and adverse events of special interest (AEoSI).

Secondary outcomes regarding pharmacokinetic/-dynamic profiles, cost-effectiveness, and predictors of successful DR were also formulated in the study protocol and will be addressed in consecutive manuscripts.

### Statistical analysis

The initial protocol was designed in 2018, therefore, the final analyses were adapted to be in accordance with current guidelines and current statistical best practice. A detailed and fully transparent description of all statistical methods is provided below with an extension in Supplementary Appendix 1 (including clarification of all adaptations to the initial protocol).

The non-inferiority margin of 15.0%, for the difference in the incidence proportion in persistent flares, was based on previous literature,^1^^,^^13^ and clinical grounds. Extensive rationale can be found elsewhere.^11^ Based on this margin, one-sided testing (α = 0.025; 1–β = 0.8), and a 2:1 randomisation ratio of DR to UC, a total of 222 patients were required to reject the null hypothesis of inferiority. Accounting for the anticipated dropout rate, the total sample size increased to 244 patients.

Baseline and treatment characteristics were summarised using descriptive statistics expressed as frequencies (% (n)), mean with standard deviation (±SD), and median with interquartile range [IQR]. The primary outcome—incidence proportion of persistent flares at 18 months—was summarised for each treatment group as marginal risks (proportions). Treatment effects were expressed as risk differences. Marginal risks and risk differences were estimated using marginal standardisation based on a generalised linear model for the binary outcome with an identity contrast. The model included treatment group and stratification factor (biologic), and estimates were standardised over the empirical covariate distribution of the study population. Risk differences and corresponding 95% CI were estimated using the delta method, with variance estimation accounting for the stratification factor. Marginal risks for each treatment group were obtained from the same outcome model by assigning the treatment indicator to each level sequentially, and averaging predicted probabilities across all participants. Correction for stratification factor was not explicitly mentioned in the pre-published study protocol but is a general rule and therefore applied for the primary outcome.^14^ However, as a sensitivity analysis of the primary outcome, unadjusted risk differences and corresponding 95% CI were also shown, and calculated using the Wilson-Newcombe method. In addition to the 15.0% non-inferiority margin, the analyses of the primary outcome were also performed for 10.0% and 5.0% margins as post-hoc sensitivity analyses. Of note, the 5.0% margin was found too conservative, not commonly acceptable like the 10.0% and 15.0% margins, and would require an unfeasibly large sample size. Time to persistent flare was visualised using a Kaplan–Meier curve. An intention-to-treat (ITT) analysis was performed as primary analysis. In the initial protocol designed in 2018, the per-protocol (PP) analysis was set as primary method as it was regarded as most conservative. However, according to current EMA guidelines on non-inferiority studies, and evolving insights on estimands, an ITT analysis is preferred as primary analysis as a PP approach could be vulnerable to bias, and is presented as such.^15^^,^^16^ Under specific circumstances, the ITT approach might still attenuate results towards non-inferiority, therefore, two PP analyses (named PP1 and PP2 that differ in population, see below) were also presented.^17^ Supplementary Table S1 shows a detailed overview of intercurrent events (i.e. the events after randomisation, including lost to follow-up and protocol deviations) and how they were handled per analysis (ITT, PP1, PP2). In the ITT analysis, patients were analysed according to treatment allocation, and intercurrent events were accepted as part of real-world practice. In the PP1 analysis, patients in the DR group were excluded from analysis from the point of an intercurrent event onward, and patients in the UC group were excluded after any changes to their standard dose. The PP2 analysis reflects a broader interpretation of the protocol which is more in line with the pragmatic design of the study; patients with DR were allowed to make a new DR attempt after reescalation, and to switch to another biologic if the DR protocol was followed prior to switching, and patients in the UC group were allowed to modify their treatment as part of usual care (Supplementary Table S1). Although patients were excluded from the PP analyses from the point of an intercurrent event onward, they did complete all study procedures. Occurrence of intercurrent events was summarised and reported. Regarding missing data, in the ITT analysis, multiple imputation (ITT_MI) was performed for the primary outcome, and last observation carried forward on all missing time points (ITT_LOCF) for both primary and secondary outcomes. In both the PP analyses, LOCF was performed for both primary and secondary outcomes for missing data until occurrence of an intercurrent event (Supplementary Table S1). The extent and nature of missing data were reported. Although our initial protocol specified LOCF for imputing missing data, we have opted to also include MI to better account for uncertainty, and reduce bias that LOCF may introduce.^18^

In the DR group, the proportion of patients with successful DR after 12 and 18 months was reported descriptively for the total population, for subgroups by drug class, and individual drugs. The between-group difference in PASI scores at each three-monthly time point was analysed by an unpaired t-test. Analysis of covariance (ANCOVA) was performed to test between-group differences in PASI and DLQI scores at 12 and 18 months, adjusted for baseline variables (gender, body mass index, disease duration, concomitant psoriatic arthritis (PsA), and biologic naivety). The difference in the proportion of patients with initiation of other anti-psoriatic medications between DR and UC was analysed descriptively.

Serious adverse events (SAEs), adverse events (AE), and adverse events of special interest (AEoSI) were summarised as proportions (without imputing data for missing time points), and rate ratios (RR) with 95% CI. AEoSI included infection, malignancy, worsening of existent PsA, new-onset PsA, musculoskeletal complaints, hypersensitivity reaction, autoimmune disease, pustular psoriasis, psychologic complaints, CVA, and myocardial infarction. RRs were compared between groups using the Wald test. Additionally, SAEs deemed specifically related to DR itself, as assessed by two reviewers (CR and JR), were analysed.

Statistical analyses were performed with SPSS software, version 29.0 (SPSS Inc), and R, version 4.5.2. No data monitoring committee was installed; the data was monitored by external data-monitors according to local guidelines.

### Role of the funding source

This was a company independent study, funded by ZonMw (The Netherlands Organization for Health Research and Development) and KCE (The Belgian Health Care Knowledge Centre). The funders of the study had no role in study design, data collection, data analysis, data interpretation, or writing of the report.

## Results

**Fig. 1:**
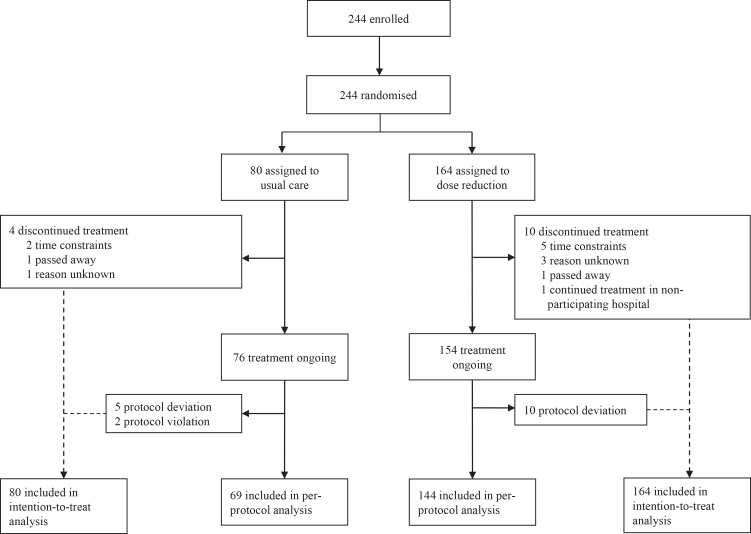
**Study flowchart.** The per-protocol analysis refers to the per-protocol 1 (PP1) analysis.

Patients were included from June 30, 2020, to September 14, 2023, across 19 dermatology departments in the Netherlands and Belgium with last patient last visit on January 30, 2025. A total of 244 patients was included (mean age 51 (±15) years; 67.0% male) with 164 patients randomised to DR and 80 to UC. At baseline 47.0% (115/244) of patients used an IL17i, and 53.0% (129/244) an IL23i. Baseline characteristics were comparable for both the DR and UC group. In the total group, PASI and DLQI scores were low with a median score of 0.0 [1.1] and 0.0 [1.0], respectively (Table 1), with 74.6% (182/244) of patients having PASI 0–1, and 84.8% (207/244) DLQI 0–1. After 18 months, 14 patients were lost to follow-up due to time constraints (N = 7), continued treatment in a non-participating hospital (N = 1), death (N = 2), or for unspecified reasons (N = 4). In two patients a protocol violation had occurred (N = 1 was included incorrectly with DLQI >5 at inclusion, N = 1 developed a conflict of interest after inclusion), and in 15 patients a protocol deviation had occurred (N = 5 continued DR while PASI >5 and/or DLQI >10, N = 3 started DR while randomised to UC of which one patient directly used a reduced dose after baseline visit, N = 1 stopped DR but reduced the dose again in subsequent visits, N = 1 used a systematically longer interval than described in the DR protocol and eventually stopped the biologic at own initiative, N = 1 stopped biologic at own initiative, N = 4 switched biologic). All 244 patients were included in the ITT_MI and ITT_LOCF analyses (DR N = 164, UC N = 80). For 213 patients in the PP1 analysis (DR N = 144, UC N = 69) (Fig. 1), and 221 patients in the PP2 analysis (DR N = 148, UC N = 73) complete study follow-up was available to assess the primary outcome. Note that some secondary analyses included more patients as they were left out from PP analyses from the point of occurrence of an intercurrent event. Over 18 months study follow-up, a total of 141/1708 visits were missing (8.3% of total planned visits; 8.3% for PASI, 8.6% for DLQI). The total number of visits performed additionally to the planned visits over 18 months was very low and comparable between the DR (4 additional visits) and UC group (2 additional visits) considering the 2:1 randomisation.

**Table 1:** Baseline characteristics of the total BeNeBio study population, and split for DR and UC group.

	Study population (N = 244)	Dose reduction group (N = 164)	Usual care group (N = 80)
Age (years)	51 ± 15	49 ± 15	54 ± 15
Male	67.0% (163)	65.0% (106)	71.0% (57)
BMI	27.1 [5.8]	26.9 [5.5]	27.6 [6.7]
Age at onset of psoriasis	27 ± 16	26 ± 16	30 ± 16
Disease duration (years)	21 [20]	21 [21]	22 [19]
PASI	0.0 [1.1]	0.0 [0.9]	0.05 [1.2]
DLQI	0.0 [1.0]	0.0 [1.0]	0.0 [1.0]
Biologic naivety	47.0% (114)	50.0% (82)	40.0% (32)
**Number of patients using an IL17 inhibitor**			
Secukinumab	14.0% (35)	15.0% (24)	14.0% (11)
Ixekizumab	25.0% (62)	25.0% (41)	26.0% (21)
Brodalumab	6.0% (15)	6.0% (10)	6.0% (5)
Bimekizumab	1.0% (3)	1.0% (2)	1.0% (1)
**Number of patients using an IL23 inhibitor**			
Guselkumab	30.0% (72)	29.0% (48)	30.0% (24)
Risankizumab	21.0% (51)	21.0% (35)	20.0% (16)
Tildrakizumab	3.0% (6)	2.0% (4)	2.5% (2)
**Concomitant use of conventional systemic therapy at start of study**			
Methotrexate	1.6% (4)	1.2% (2)	2.5% (2)
Acitretin	0.4% (1)	0.0% (0)	1.3% (1)
**Number of previously used treatments per patient**			
Conventional systemic	2.0 [1.0]	2.0 [1.0]	2.0 [1.0]
Biologic/SMI	1.0 [1.0]	0.5 [1.0]	1.0 [1.8]
**Comorbidities of interest**			
Cardiovascular disease	34.0% (84)	33.0% (54)	37.5% (30)
Depression/anxiety	15.0% (36)	18.0% (29)	9.0% (7)
Rheumatologic condition—concomitant PsA	14.0% (34)	13.0% (21)	16.0% (13)
Rheumatologic condition—not PsA	7.0% (18)	7.0% (11)	9.0% (7)
Diabetes mellitus (type 1/2)	11.0% (27)	12.0% (19)	10.0% (8)
Inflammatory bowel disease	2.0% (5)	2.0% (3)	2.5% (2)
Malignancy	3.0% (8)	2.0% (4)	5.0% (4)
Non-melanoma skin cancer	0.8% (2)^a^	1.0% (2)^a^	0.0% (0)

Means with standard deviations (±SD), number of patients (n) with percentages (%), and medians with interquartile range [IQR].

BMI, Body Mass Index; PASI, Psoriasis Area and Severity Index; DLQI, Dermatology Life Quality Index; SMI, Small Molecule Inhibitor; PsA, psoriatic arthritis.

a Both cases concerned basal cell carcinoma.

**Fig. 2:**
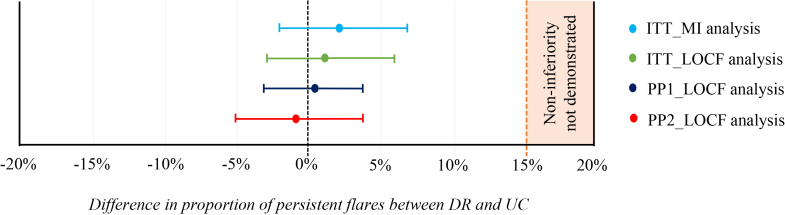
**The difference in proportion of persistent flares between dose reduction (DR) and usual care (UC) after 18 months for all analyses.** Error bars indicate 95% confidence intervals. The orange line at 15.0% shows the non-inferiority margin set at 15.0%. ITT, intention-to-treat; PP, per-protocol; MI, multiple imputation; LOCF, last observation carried forward.

Regarding the primary outcome, the incidence proportion of persistent flares was 4.0% in the DR, and 1.6% in the UC group after 18 months in the ITT_MI analysis. The difference in the proportion of persistent flares between DR and UC, after correction for stratification factor, was 2.4% (95% CI −2.0% to 6.8%) indicating non-inferiority of the primary outcome (Fig. 2). The ITT_LOCF analysis showed incidence proportions of 3.7% (6/164 patients) and 2.5% (2/80 patients) in the DR and UC group, respectively, with a difference in proportion of persistent flares of 1.2% (95% CI −3.3% to 5.6%). Non-inferiority of DR compared to UC was also shown in both PP analyses (Fig. 2). Sensitivity analysis with unadjusted risk difference confidence intervals also showed non-inferiority of DR compared to UC in both the ITT_LOCF and ITT_MI analysis, as well as in both PP analyses (ITT_LOCF: 1.2% (95% CI −6.2% to 6.1%), ITT_MI: 2.4% (95% CI −5.6% to 7.2%), PP1: 0.6% (95% CI −7.0% to 5.2%), PP2: −0.7% (95% CI −8.6% to 4.1%). A sensitivity analysis visualising non-inferiority with other margins can be found in Supplementary Figure S2.

The course of PASI of patients with persistent flares in ITT_LOCF, PP1, and PP2 is shown in Supplementary Table S2, and time to persistent flares is visualised in Kaplan–Meier curves in Supplementary Figure S3. Analysis of the incidence of short disease flares (PASI >5 at one timepoint) showed that 15.9% (26/164) of patients in the DR group had at least one short disease flare, and 6.3% (5/80) in the UC group (Supplementary Table S3; ITT_LOCF). The PP1 and PP2 analyses showed similar courses of persistent flares (Supplementary Table S2), and similar incidences of short disease flares (Supplementary Table S3).

Regarding the proportions of successful DR, 125/164 patients (76.2%) achieved successful DR after 12 months, and 120/164 patients (73.2%) after 18 months (Supplementary Table S4; ITT_LOCF). At both timepoints, an additional three patients also used a lower dose but did not achieve successful DR as PASI scores were >5. After 12 months, a total of 34/164 patients (20.7%) successfully used 67.0% of their original dose (step 1 of DR protocol) and 91/164 patients (55.5%) used 50.0% of their original dose (step 2 of DR protocol). After 18 months, a total of 37/164 patients (22.6%) successfully reduced their dose to step 1, and 83/164 patients (50.6%) to step 2. In the DR group, 61.0% (47/77) of the patients with an IL17i, and 83.9% (73/87) of the patients with an IL23i, achieved successful DR after 18 months (Supplementary Table S5; ITT_LOCF). The proportion of patients with successful DR was similar in the PP1 and PP2 analyses (Supplementary Table S4), also when split per biologic (Supplementary Table S5).

**Fig. 3:**
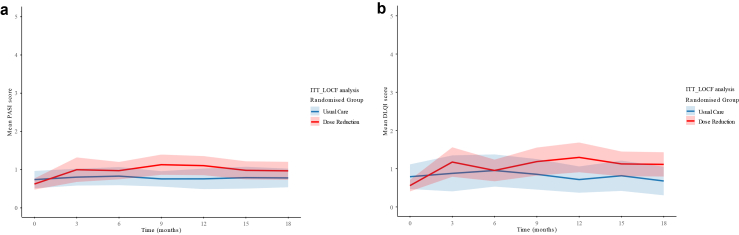
**Course of mean PASI and DLQI scores over 18 months study follow-up in the intention-to-treat analysis with last observation carried forward (ITT_LOCF).** The mean PASI scores (a) and DLQI scores (b) with 95% confidence interval, for both DR and UC group, are shown per visit. DR, dose reduction; UC, usual care; PASI, Psoriasis Area and Severity Index; DLQI, Dermatology Life Quality Index.

The course of mean PASI and DLQI over 18 months study follow-up for the DR and UC group in the ITT_LOCF analysis are presented in Fig. 3. The mean PASI after 18 months was 1.0 (±1.5) in the DR, and 0.8 (±1.1) in the UC group. The mean DLQI after 18 months was 1.1 (±2.1) in the DR, and 0.7 (±1.7) in the UC group. The PP1 and PP2 analyses showed a similar course of mean PASI and DLQI (Supplementary Figure S4). In the ITT_LOCF analysis, mean PASI was statistically significant higher in DR compared to UC at visit month 9 only with 0.4 (p = 0.0280). In the PP1 and PP2 analyses, at none of the three-monthly timepoints a statistically significant difference in mean PASI between DR and UC was seen (Supplementary Table S6). At month 12 and month 18, ANCOVA showed no significant differences in mean PASI and DLQI between DR and UC in ITT_LOCF (Table 2). The PP1 and PP2 analyses also showed no significant differences at month 12 and 18 (Supplementary Table S7).

**Table 2:** Analysis of covariance (ANCOVA) of PASI and DLQI between dose reduction (DR) and usual care (UC) at month 12 and month 18 for the intention-to-treat analysis with last observation carried forward (ITT_LOCF).

ITT_LOCF	DR mean (±SD)	UC mean (±SD)	p-value
PASI M12	1.1 (±1.5)	0.7 (±1.5)	0.0510
PASI M18	1.0 (±1.4)	0.7 (±1.4)	0.1890
DLQI M12	1.3 (±2.3)	0.7 (±2.2)	0.0560
DLQI M18	1.1 (±1.9)	0.6 (±2.0)	0.0700

PASI, Psoriasis Area and Severity Index; DLQI, Dermatology Life Quality Index; SD, standard deviation; M, visit month.

The number of patients that used concomitant methotrexate or acitretin at baseline was 2/164 (1.2%) in the DR, and 3/80 (3.8%) in the UC group in ITT_LOCF. During the study, 3/164 patients (1.8%) in the DR group initiated methotrexate of which one patient stopped methotrexate before end of the study. In the UC group, 1/80 patients (1.3%) initiated methotrexate during the study and stopped before end of study as well. Acitretin was initiated in 3/164 patients (1.8%) in the DR group of which one patient stopped before end of study. In total, 3/164 patients in the DR group (1.8%) switched to another biologic: one patient due to increased psoriasis activity (with PASI 3.7, DLQI 5.0 but no (persistent) flare), one patient with newly developed PsA, and one patient due to pregnancy plans. In the UC group, 1/80 patients (1.3%) switched to another biologic due to increased psoriasis activity of psoriasis (with PASI 5.5, DLQI 6.0 but no persistent flare). One patient in each group stopped biologic treatment on their own initiative (DR 0.6% (1/164), UC 1.3% (1/80)). The use of concomitant anti-psoriatic treatments was similar in the PP1 and PP2 analyses.

In the DR group, a total of 11 SAE was reported in 9/164 patients, and in the UC group, 4 SAE were reported in 3/80 patients in the ITT analysis. None of the 11 reported SAE in the DR group were deemed related to DR. Table 3 shows the rates and RR of the SAE and AEoSI in the ITT analysis. The difference in total number of SAE between DR and UC was not statistically significant (RR 1.3 (95% CI 0.4–4.1)). Per AEoSI category, no RR was statistically significant for DR versus UC. In the DR group, 4/164 (2.4%) patients were diagnosed with new-onset PsA during the study (1.7 (95% CI 0.5–3.7) per 100 observation years), and none in the UC group. Worsening of pre-existent PsA occurred in 5/164 (3.0%) and 4/80 (5.0%) patients in respectively the DR, and UC group (RR 0.6 (95% CI 0.2–2.2)). In the PP1 and PP2 analyses, the safety profile for DR versus UC was similar (Supplementary Table S8).

**Table 3:** Safety of dose reduction (DR) versus usual care (UC) in the intention-to-treat analysis (ITT).

ITT	Event rate per 100 observation years (95% CI)		Rate ratio (95% CI) for DR versus UC
	DR group (N = 164)	UC group (N = 80)	
Total SAE	4.7 (2.3–7.8)	3.6 (1.0–7.9)	1.3 (0.4–4.1)
Total AE	112.6 (99.5–126.6)	125.8 (105.8–147.5)	0.9 (0.7–1.1)
Total AEoSI	72.7 (62.2–84.0)	83.6 (67.5–101.4)	0.9 (0.7–1.1)
Infection^a^	47.6 (39.2–56.8)	60.2 (46.7–75.5)	0.8 (0.6–1.1)
Malignancy^a^	1.7 (0.5–3.7)	*NA*	*NA*
Worsening pre-existent PsA^a^	2.1 (0.7–4.4)	3.6 (1.0–7.9)	0.6 (0.2–2.2)
PsA (new diagnosis)^a^	1.7 (0.5–3.7)	*NA*	*NA*
Musculoskeletal complaints (excluding PsA or trauma)^a^	15.3 (10.7–20.7)	15.3 (8.9–23.3)	1.0 (0.6–1.8)
Hypersensitivity reaction to biologic (including injection site reaction)^a^	1.3 (0.3–3.1)	0.9 (0.0–3.3)	1.4 (0.1–13.6)
Autoimmune disease (excluding PsA)^a^	0.4 (0.0–1.6)	*NA*	*NA*
Pustular psoriasis^a^	0.4 (0.0–1.6)	*NA*	*NA*
Psychologic complaints^a^	0.4 (0.0–1.6)	0.9 (0.0–3.3)	0.5 (0.0–7.6)
CVA^a^	0.9 (0.1–2.4)	1.8 (0.2–5.0)	0.5 (0.1–3.4)
Myocardial infarction^a^	0.9 (0.1–2.4)	0.9 (0.0–3.3)	0.9 (0.1–10.4)
Neurologic event (without musculoskeletal or CVA)	2.6 (0.9–5.0)	1.8 (0.2–5.0)	1.4 (0.3–7.0)
Gastrointestinal (including hepatologic)	3.8 (1.7–6.7)	4.5 (1.5–9.2)	0.9 (0.3–2.5)
Gyn-urological	3.0 (1.2–5.5)	*NA*	*NA*
Unexpected reaction to vaccine	0.4 (0.0–1.6)	*NA*	*NA*
Other	30.2 (23.6–37.6)	35.9 (25.7–47.9)	0.8 (0.6–1.2)

Safety is shown as event rate per 100 observation years and the rate ratio with 95% confidence intervals (CI). SAE, serious adverse events; AE, adverse events; AEoSI, adverse events of special interest; PsA, psoriatic arthritis; CVA, cerebrovascular event; NA, not applicable.

a Category included as AEoSI.

## Discussion

This multicentre, pragmatic, open-label, randomised controlled trial showed that this stepwise dose reduction (DR) strategy, including timely reescalation, of IL17i and IL23i in patients with stable plaque psoriasis was non-inferior to usual care (UC) regarding persistent disease flares. At the end of the study, successful DR was achieved by 73.2% of the patients in the DR group; this percentage was higher in IL23i users compared to IL17i users (83.9% resp. 61.0%). During the study, mean PASI and DLQI scores remained low in both the DR and UC group. No SAEs related to DR were detected. These results show that DR of the investigated biologics is an effective approach to manage the disease activity of patients with psoriasis with low or absent disease activity.

In both the DR and the UC cohort, all patients with a persistent flare re-established a PASI <5 upon returning to the previous effective dose or the standard dose, except for one patient in each group and one additional patient in the DR cohort who stopped treatment on his own initiative. The proportion of patients with a short disease flare was low in both groups, but higher in the DR group compared to the UC group (15.9% and 6.3% respectively). We can conclude from these numbers that there is always a risk on a short disease flare as psoriasis is a chronic, unpredictable disease, even when using an effective biologic in the standard dose (UC). Additionally, short flares are inherent to this DR strategy as the limits of the dose–response are being explored. The 3-monthly follow-up visits ensured timely counselling, which is important to avoid persistent flares. Reassuringly, as shown by Supplementary Figure S5, short disease flares did not often lead to high PASI scores. In addition, visualisation of time until first dose reescalation shows that we applied timely reescalation throughout the study, to prevent patients from developing persistent flares (Supplementary Figure S6). The finding that an adequate treatment response can generally be restored within a modest time frame after a flare during DR is in line with previous DR studies on adalimumab, etanercept, and ustekinumab.^19^, ^20^, ^21^, ^22^, ^23^

A previous randomised trial on a DR strategy of adalimumab, etanercept, and ustekinumab showed that 53.0% of patients had successfully lowered their dose after one year of study follow-up.^1^ Here we show that DR was even more successful in patients with psoriasis treated with an IL17i or IL23i (76.2% after 12 months, and 73.2% after 18 months). It is important to note that the baseline median PASI and DLQI were lower (0.0 [1.1] and 0.0 [1.0], respectively) in this present study. The high effectiveness of IL17i and IL23i might also contribute to the higher proportion of patients with successful DR in this present study compared to the previous trial. Interestingly, some baseline characteristics of patients with unsuccessful DR differed from patients with successful DR, for instance, slightly more patients were biologic naive, and less had a cardiovascular disease in the successful versus unsuccessful DR group (see Supplementary Table S9). In depth analysis on the drivers for successful DR will separately be analysed combined with the pharmacological outcomes.

Although the study was not powered for subgroup analyses per biologic, we descriptively compared the group of patients treated with IL17i to those with IL23i, and report a higher proportion of successful DR in the IL23i group (61.0% versus 83.9%). To draw definitive conclusions per biologic, large studies per drug should be performed. A previous randomised withdrawal study already showed that IL23 inhibition with risankizumab provided durable disease control with the possibility of withdrawal in a subpopulation of patients.^24^ In addition, the GUIDE study showed non-inferiority of interval prolongation of guselkumab in super-responders compared to standard dose.^5^

Regarding the chosen inclusion criteria of disease activity (PASI ≤5) and quality of life (DLQI ≤5), these were the prevailing treatment goals as stated by Mrowietz et al. (2011), and the Euroguiderm guideline at the start of the study design in 2018.^25^ Currently, a lower value for PASI and/or DLQI would be preferable, as also stated in a recent eDelphi study, where a minimal residual PASI <3 was preferred as definition of disease control.^26^ Reassuringly, in the present study, the vast majority of patients (74.6%) had a very low PASI (0–1) at baseline, and the very few with a PASI >1 and ≤ 5 always had minimal impact on their lives. The composition of our study population might indicate that this DR strategy is predominantly successful in patients with a very low PASI at start of DR. In addition, mean PASI and DLQI remained very low during the study in both the DR and UC group. Only in the ITT_LOCF analysis, there was a statistically significant difference in mean PASI between DR and UC at month 9, however, the small difference of 0.4 was not deemed clinically relevant, and was not seen at the subsequent time points until the end of study at 18 months.

Even though there were no SAEs related to DR, it is important to note that there were 4 cases of new-onset PsA (1.7 (95% CI 0.5–3.7) per 100 observation years) in the DR, and none in the UC group. This difference could be coincidental as the UC group was also substantially smaller. Interestingly, worsening of PsA occurred more frequently in the UC than in the DR group. Also, we know from multiple studies that—even on biologics—a small part of patients can still develop PsA. For instance, the incidence rate of PsA in patients with psoriasis on biologics in general ranges from an annual incidence rate of 2.4–5.5 per 100 patient-years (py), and is 1.9 (95% CI 0.6–5.7) for IL17i, and 1.3 (95% CI 0.3–5.2) for IL23i per 100py.^27^^,^^28^ These incidence rates allow a cautious hypothesis that the risk of developing new-onset PsA is not increased when reducing biologic dose compared to continuing standard dose. However, long term effects and the risk on PsA should be determined in long-term follow-up DR studies.

Besides developing adverse events, the development of antidrug antibodies (ADAs) against a biologic when prolonging the injection interval has also been part of the debate regarding safety of biologic DR since interval prolongation of infliximab resulted in increased levels of ADAs.^29^ However, no (relevant) ADA development nor a significant difference in ADA levels between DR and the standard dose of adalimumab, etanercept, and ustekinumab was shown in previous studies.^3^ The development of ADAs in DR of IL17i and IL23i needs to be further explored, though, no issues regarding ADA development is expected as recent studies showed that ADA levels in IL17i and IL23i were either low (in secukinumab, brodalumab, tildrakizumab, guselkumab, ixekizumab), or relatively high but without impairing clinical response (in risankizumab, and bimekizumab), or mainly transient (in secukinumab, and brodalumab).^30^^,^^31^

In light of another secondary outcome of this trial, proactive therapeutic drug monitoring (TDM) may represent an additional approach to personalise dosing. As secondary outcome, our trial also included the development of pharmacokinetic and pharmacodynamic (PKPD) models on the collected serum drug concentration samples, and the investigation of a potential correlation between serum trough concentration levels of biologics and successful DR. These results will be reported in a separate publication. By enabling individualised dose optimisation based on patient-specific PKPD data, proactive TDM has the potential to aim for the minimal effective drug concentration in each patient. In addition, ‘on demand’ treatment is also gaining ground as a personalised dosing strategy and is currently being studied.^32^

It is important to address the cost-effectiveness of DR of IL17i and IL23i in patients with psoriasis, as DR can contribute to a substantial reduction of medication costs. Previous studies on DR of TNFαi, and ustekinumab showed considerable annual cost savings without significantly impacting quality of life.^2^^,^^3^ In the near future, a separate paper will elaborate on cost-effectiveness of DR, derived from our current trial.

A strength of this study is the pragmatic design, enhancing the study's external validity as it ensures that findings reflect real-world clinical practice. Although blinding was not performed for the same reason, it could potentially lead to bias in outcomes like PASI scores. This was mitigated by the performance of predefined and standardised scoring procedures (e.g. PASI assessments) by trained study personnel. In addition, where possible, assessments were conducted by the same assessor at follow-up visits to ensure internal consistency. Bias due to non-blinding could also play a role in safety reporting by patients, which was mitigated by separately also reporting on more objectifiable outcomes like SAEs related to DR. Other limitations of the study are the occurrence of missing values. However, as two different methods for analysis (ITT and PP), and two different methods to impute missing data (MI and LOCF), and both a model-based analysis with correction for stratification as well as unadjusted analyses, all showed similar affirmative results with regard to meeting the primary objective of the trial, confidence in the robustness of results is provided.^33^ The non-inferiority margin of 15.0% was based on previous literature,^1^^,^^13^ and clinical grounds. The choice of the non-inferiority margin inevitably involves a degree of arbitrariness, as it includes a judgement about what magnitude of loss of efficacy would still be considered clinically acceptable. Reassuringly, as can be seen in Supplementary Figure S2 in the present study, the DR strategy would also be considered non-inferior to UC when a margin of 10.0% would have been chosen here.

In conclusion, this first, multicentre, randomised, controlled, pragmatic trial regarding dose reduction of IL17i and IL23i by stepwise prolongation of the injection interval in patients with psoriasis who had low or absent disease activity, and a good quality of life, showed that dose reduction was non-inferior to usual care. Successful and sustained DR was achieved in a substantial proportion of patients (≈73.0%) at 18 months. These results support further implementation of a dose reduction strategy of the investigated biologics in real-world clinical practice, and highlight the potential for personalised treatment approaches in psoriasis management.

## Contributors

JvdR: conceptualisation, data curation, data verification, access to raw data, formal analysis, funding acquisition, methodology, project administration, resources, supervision, validation, visualisation, writing–original draft, writing–review & editing, and responsibility for the decision to submit for publication.

CvR: data curation, data verification, access to raw data, formal analysis, investigation, methodology, project administration, resources, validation, visualisation, writing–original draft, writing–review & editing, and responsibility for the decision to submit for publication.

RS: conceptualisation, access to raw data, funding acquisition, methodology, project administration, resources, supervision, and writing–review & editing.

AE: investigation, project administration, and writing–review & editing.

LS: conceptualisation, funding acquisition, investigation, methodology, project administration, resources, and writing–review & editing.

LvdS: conceptualisation, funding acquisition, investigation, methodology, project administration, resources, and writing–review & editing.

LG: conceptualisation, funding acquisition, investigation, methodology, project administration, resources, and writing–review & editing.

GH: formal analysis, methodology, validation, visualisation, and writing-review & editing.

FW: investigation, project administration, resources, and writing–review & editing.

TH: investigation, project administration, resources, and writing–review & editing.

PvL: investigation, project administration, resources, and writing–review & editing.

PG: investigation, project administration, resources, and writing–review & editing.

PO: investigation, project administration, resources, and writing–review & editing.

SL: investigation, project administration, resources, and writing–review & editing.

SP: investigation, project administration, resources, and writing–review & editing.

LT: investigation, project administration, resources, and writing–review & editing.

MvD: investigation, project administration, resources, and writing–review & editing.

EvdV: investigation, project administration, resources, and writing–review & editing.

IH: investigation, project administration, resources, and writing–review & editing.

JH: investigation, project administration, resources, and writing–review & editing.

AN: investigation, project administration, resources, and writing–review & editing.

LD: investigation, project administration, resources, and writing–review & editing.

MB: investigation, project administration, resources, and writing–review & editing.

SD: investigation, project administration, resources, and writing–review & editing.

HB: investigation, project administration, resources, and writing–review & editing.

JL: conceptualisation, funding acquisition, methodology, project administration, resources, supervision, validation, writing–review & editing, and responsibility for the decision to submit for publication.

EdJ: conceptualisation, funding acquisition, methodology, project administration, resources, supervision, validation, writing–original draft, writing–review & editing, and responsibility for the decision to submit for publication.

## Data sharing statement

One year after publication, the pseudonymized, individual participant data that underlie the results reported in this article (text, tables, figures, and appendices), will be shared to those whose proposed use of the data has been approved by an independent review committee/medical ethical committee identified for this purpose. Data will be stored in a repository of one of the main investigating centres (Radboud University Medical Centre) and will be available indefinitely. Data requests can be send to the corresponding author.

## Declaration of interests

JvdR received funding for research and for mentoring program (IPC), funding from NWO (the Dutch Research Council) for participating in the dermatology consortium; works as a coordinator for the BioCAPTURE registry, the registry was sponsored by AbbVie, Janssen, Lilly, Almirall, UCB, and Leo Pharma; received fees as advisory board member, and speaking fees, from Almirall, Lilly, Leo Pharma, BMS, AbbVie, Zoetis, UCB, and Janssen; received a travel grant from the European Academy of Dermatology and Venereology (EADV), and was sponsored by Janssen for visiting scientific symposium; works as a sub-investigator in trials of Almirall, and AstraZeneca. All funding is not personal but goes to the independent research fund of the department of dermatology of Radboudumc Nijmegen, the Netherlands.

CvR was sponsored by Janssen for visiting scientific symposium; works as a sub-investigator for the PSOLAR registry by Janssen. All funding is not personal but goes to the independent research fund of the department of dermatology of Radboudumc Nijmegen, the Netherlands.

RS declares no competing interests.

AE declares no competing interests.

LS has received paid speaker fees by AbbVie, Novartis and Janssen. All fees were paid to the Ghent University (Hospital) scientific accounts.

LvdS carried out clinical trials for Almirall, Janssen, and Novartis, and received speaker fees from Janssen. All funding is not personal but goes to the independent Research Fund of the Department of Dermatology of the Radboud University Medical Centre, Nijmegen, Netherlands.

LG is currently employed as scientist at argenx, received speaker fees from Leo Pharma, Kintu, AbbVie, Novartis, R-Biopharm, received teaching fees from Kintu and Erasmus College, and received writing fees from Royal Belgian Society for Dermatology and Venerology.

GH declares no competing interests.

FW declares no competing interests.

TH reports consultancy from AbbVie, Almirall, Amgen, Boehringer Ingelheim, Bristol Myers Squibb, Celgene, Janssen, Leo Pharma, Eli Lilly, Novartis, Sandoz, and UCB Pharma; speaker fees from AbbVie, Almirall, Amgen, Biogen, Bristol Myers Squibb, Celgene, Janssen, Leo Pharma, L’Oréal, Eli Lilly, Novartis, Sanofi, and UCB Pharma; research funding from AbbVie, Almirall, Amgen, Janssen, Leo Pharma, Eli Lilly, Novartis, and UCB Pharma.

PvL received funding from Wyeth for research, and carried out clinical trials for Abbott and Janssen-Cilag; received speaking and consulting fees from Wyeth, and Schering-Plough; received reimbursement for attending a symposium from Schering-Plough, and Pfizer; attended advisory boards for AbbVie, Leo Pharma, Novartis, and UCB. Starting from 2019, PvL has no conflicts of interest to declare.

PG participated in advisory boards/data safety monitoring boards, and/or received consulting fees, and/or speaking fees, and/or travel support from Pfizer, MSD, AbbVie, Janssen, Serono, Leo, Novartis, UCB, Amgen, Eli-Lilly, Galderma, BMS, Meda, Viatris, Maruho, Flen, Menarini, PellePharm, Boehringer, Pierre Fabre, Sandoz, InCyte EG, and Almirall.

PO declares no competing interests.

SL received consulting fees from Eli Lilly, Janssen, Leo Pharma, received speaker fees from Janssen, received travel support from AbbVie, UCB, Almirall, and participated in advisory boards/data safety monitoring boards of Leo Pharma, and Lilly.

SP declares no competing interests.

LT declares no competing interests.

MvD received non-personal grants from Janssen, Almirall, Third Harmonic, and Escient; received personal speaking fees from Novartis, AbbVie, Leo Pharma, Sanofi, Janssen, UCB, and BMS; received personal travel support from UCB, and J&J; and received personal fees for participating in advisory boards/data safety monitoring board of Leo Pharma, and Sanofi.

EvdV declares no competing interests.

IH was a consultant, advisory board member, and/or speaker for AbbVie, Eli Lilly, Leo Pharma, Pfizer, Regeneron, and Sanofi-Genzyme.

JH was in the advisory board/data safety monitoring board of Novartis, and in the medical advisory board of Hidradenitis Suppurativa Patient Association, and received speaking fees from AbbVie.

AN declares no competing interests.

LD declares no competing interests.

MB declares no competing interests.

SD attended advisory boards for AbbVie, Leo Pharma, Janssen, and Novartis.

HB was in the advisory board, and/or speaker, for AbbVie, UCB pharma, Galderma, Novartis, Leo pharma, Lilly, Sanofi, Jansen, Incyte, Fellos, and participated in trials from/by Leo pharma, Lilly, and Incyte.

JL has received scientific research grants from J&J, AbbVie and Pfizer; paid consultancies for scientific research from AbbVie, Almirall, Argenx, BMS, J&J, Pfizer, Leo Pharma, Novartis and UCB; and carried out clinical trials for Janssen-Cilag, Merck Serono, Amgen, Pfizer, AbbVie, Celgene, Moonlake Ther, Regeneron and Novartis.

EdJ received research grants for the department of dermatology of the Radboud University Medical Centre Nijmegen, the Netherlands from AbbVie, Almirall, J&J, Leo Pharma, Lilly, and UCB, acted as consultant and/or paid speaker for and/or participated in research sponsored by companies that manufacture drugs used for the treatment of psoriasis including AbbVie, Almirall, Boehringer-Ingelheim, Bristol Myers Squibb, J&J, Leo Pharma, Lilly, and UCB. All funding is not personal but goes to the Institution Radboud University Medical Centre Nijmegen, the Netherlands.

## Appendix A Supplementary data

Supplementary Appendix 1

Study Protocol BeNeBio trial

Captions
