# Supplementary material for: Dose Reduction of IL17 and IL23 Inhibitors in Psoriasis (BeNeBio study): An International, Pragmatic, Multicentre, Randomised, Controlled, Non-Inferiority Trial
Source: Lancet Reg Health Eur. 2026 Jun 1;67:101721. doi: 10.1016/j.lanepe.2026.101721 (PMC13453948; doi:10.1016/j.lanepe.2026.101721)
Supplement: Supplementary file 2 [file mmc1.docx]

**Table of contents Supplementary Tables and Figures**

[Supplementary Table 1: Overview of how intercurrent events are handled per analysis	2](#_Toc227848766)

[Supplementary Table 2: Course of persistent flares	3](#_Toc227848767)

[Supplementary Table 3: Incidences of short disease flares	4](#_Toc227848768)

[Supplementary Table 4: Proportion of patients with successful dose reduction	5](#_Toc227848769)

[Supplementary Table 5: Proportions of patients with successful dose reduction split for biologic	6](#_Toc227848770)

[Supplementary Table 6: Results of the unpaired t-test of the PASI scores	9](#_Toc227848771)

[Supplementary Table 7: Analysis of covariance (ANCOVA) of PASI and DLQI scores	10](#_Toc227848772)

[Supplementary Table 8: Safety of dose reduction (DR) versus usual care (UC)	11](#_Toc227848773)

[Supplementary Table 9: Baseline characteristics of the patients with successful, and unsuccessful DR	12](#_Toc227848774)

[Supplementary Figure 1: Overview of the dose reduction strategy and study measures according to the study protocol	13](#_Toc227848775)

[Supplementary Figure 2: Sensitivity analysis with additional non-inferiority margins	14](#_Toc227848776)

[Supplementary Figure 3: Kaplan-Meier curve showing time to persistent flare	15](#_Toc227848777)

[Supplementary Figure 4: Course of mean PASI and DLQI scores	17](#_Toc227848778)

[Supplementary Figure 5: Course of PASI scores on individual patient level	19](#_Toc227848779)

[Supplementary Figure 6: Kaplan-Meier curve showing time to first dose reescalation	21](#_Toc227848780)

Supplementary Table 1: Overview of how intercurrent events are handled per analysis***.***

|  | **ITT** | | **PP1** | | **PP2** | |
| --- | --- | --- | --- | --- | --- | --- |
|  | **DR** | **UC** | **DR** | **UC** | **DR** | **UC** |
| **Deviation from DR protocol: PASI >5 and/or DLQI >10 and patient remained on DR** | Included *(all data remain in ITT)* | *NA* | Excluded *(data are excluded from the time of intercurrent event)* | *NA* | Excluded  *(data are excluded from the time of intercurrent event)* | *NA* |
| **New DR attempt after returning to the standard or (previous) effective dose (reescalation of the dose)** | Included *(all data remain in ITT)* | Included *(all data remain in ITT)* | Excluded *(data are excluded from the time of intercurrent event)* | Excluded  *(data are excluded from the time of intercurrent event)* | Included *(all data remain in PP2)* | Included *(all data remain in PP2)* |
| **Start DR while randomised to UC** | *NA* | Included *(all data remain in ITT)* | *NA* | Excluded  *(data are excluded from the time of intercurrent event)* | *NA* | Included *(all data remain in PP2)* |
| **Dropout (lost to follow-up)** | Included *(all data are imputed until M18)* | Included *(all data are imputed until M18)* | Excluded  *(data are excluded from the time of intercurrent event)* | Excluded  *(data are excluded from the time of intercurrent event)* | Excluded  *(data are excluded from the time of intercurrent event)* | Excluded  *(data are excluded from the time of intercurrent event)* |
| **Switch of biologic** | Included *(all data remain in ITT)* | Included *(all data remain in ITT)* | Excluded *(data are excluded from the time of intercurrent event)* | Excluded  *(data are excluded from the time of intercurrent event)* | Included *(all data remain in PP2 but only if protocol was followed prior to switch)* | Included *(all data remain in PP2)* |
| **Withdrawal from/stop of biologic** | Included *(all data remain in ITT)* | Included *(all data remain in ITT)* | Excluded  *(data are excluded from the time of intercurrent event)* | Excluded  *(data are excluded from the time of intercurrent event)* | Excluded  *(data are excluded from the time of intercurrent event)* | Included *(all data remain in PP2)* |
| **Systematically longer / shorter injection interval than protocol without medical reason** | Included *(all data remain in ITT)* | Included *(all data remain in ITT)* | Excluded  *(data are excluded from the time of intercurrent event)* | Excluded  *(data are excluded from the time of intercurrent event)* | Excluded  *(data are excluded from the time of intercurrent event)* | Included *(all data remain in PP2)* |

*Supplementary Table 2: Course of persistent flares* **for the dose reduction (DR) and the usual care (UC) group in the intention-to-treat analysis with last observation carried forward (ITT_LOCF), per protocol 1 analysis (PP1), and per protocol 2 analysis (PP2).**

| **ITT_LOCF** | | | | | | | | |
| --- | --- | --- | --- | --- | --- | --- | --- | --- |
|  | **PASI score** | | | | | | | **Mean (**±**SD) PASI score of the persistent flare PASIs (orange marked scores)** |
|  | **M0** | **M3** | **M6** | **M9** | **M12** | **M15** | **M18** |  |
| **Dose reduction** | | | | | | | | |
| ***Patient 1*** | 4·8 | 6·9 | 9·2 | 6·0 | 0·8 | 0·8 | 4·1 | 7·4 (±1·7) |
| ***Patient 2*** | 0·0 | 2·8 | 5·6 | 6·9 | 3·5 | 0·9 | 1·5 | 6·3 (±0·9) |
| ***Patient 3*** | 0·8 | 3·3 | 3·3 | 5·5 | 10·3 | 10·3 | 5·9 | 8·0 (±2·7) |
| ***Patient 4*** | 0·0 | 6·9 | 4·8 | 7·2 | 7·6 | 0·0 | 5·6* | 7·4 (±0·3) |
| ***Patient 5*** | 2·8 | 2·6 | 5·6 | 8·2 | 3·8 | 4·6 | 2·4 | 6·9 (±1·8) |
| ***Patient 6*** | 0·8 | 1·4 | 1·4 | 4·0 | 6·1 | 7·0 | 7·0** | 6·7 (±0·5) |
| **Usual care** | | | | | | | | |
| ***Patient 1*** | 1·7 | 3·2 | 0·8 | 0·3 | 7·5 | 7·2 | 1·6 | 7·4 (±0·2) |
| ***Patient 2*** | 1·6 | 3·0 | 5·2 | 2·6 | 4·0 | 5·5 | 5·5 | 5·5 (±0·0) |
| **PP1** | | | | | | | | |
|  | **PASI score** | | | | | | | **Mean (**±**SD) PASI score of the persistent flare PASIs (orange marked scores)** |
|  | **M0** | **M3** | **M6** | **M9** | **M12** | **M15** | **M18** |  |
| **Dose reduction** | | | | | | | | |
| ***Patient 1*** | 4·8 | 6·9 | 9·2 | 6·0 | 0·8 | 0·8 | 4·1 | 7·4 (±1·7) |
| ***Patient 2*** | 0·0 | 2·8 | 5·6 | 6·9 | 3·5 | 0·9 | 1·5 | 6·3 (±0·9) |
| ***Patient 3*** | 0·8 | 3·3 | 3·3 | 5·5 | 10·3 | 10·3 | 5·9 | 8·0 (±2·7) |
| **Usual care** | | | | | | | | |
| ***Patient 1*** | 1·7 | 3·2 | 0·8 | 0·3 | 7·5 | 7·2 | 1·6 | 7·4 (±0·2) |
| **PP2** | | | | | | | | |
|  | **PASI score** | | | | | | | **Mean (**±**SD) PASI score of the persistent flare PASIs (orange marked scores)** |
|  | **M0** | **M3** | **M6** | **M9** | **M12** | **M15** | **M18** |  |
| **Dose reduction** | | | | | | | | |
| ***Patient 1*** | 4·8 | 6·9 | 9·2 | 6·0 | 0·8 | 0·8 | 4·1 | 7·4 (±1·7) |
| ***Patient 2*** | 0·0 | 2·8 | 5·6 | 6·9 | 3·5 | 0·9 | 1·5 | 6·3 (±0·9) |
| ***Patient 3*** | 0·8 | 3·3 | 3·3 | 5·5 | 10·3 | 10·3 | 5·9 | 8·0 (±2·7) |
| **Usual care** | | | | | | | | |
| ***Patient 1*** | 1·7 | 3·2 | 0·8 | 0·3 | 7·5 | 7·2 | 1·6 | 7·4 (±0·2) |
| ***Patient 2*** | 1·6 | 3·0 | 5·2 | 2·6 | 4·0 | 5·5 | 5·5 | 5·5 (±0·0) |

Supplementary Table 3: Incidences of short disease flares ***per three monthly visit for the dose reduction (DR) group and the usual care (UC) group in the intention-to-treat analysis with last observation carried forward (ITT_LOCF), per protocol 1 analysis (PP1), and per protocol 2 analysis (PP2).***

| ITT_LOCF | | | | | | |
| --- | --- | --- | --- | --- | --- | --- |
|  | **Number of short disease flares (PASI>5) in DR** | ***Mean (±SD) PASI of the patients with short disease flare in DR*** | **Number of short disease flares (PASI>5) in UC** | | ***Mean (±SD) PASI of the patients with short disease flare in UC*** | **Total** |
| M0 | 0 |  | 0 | |  | 0 |
| M3 | 3 | *11*·*9 (±8*·*7)* | 0 | |  | 3 |
| M6 | 4 | *6*·*4 (±1*·*9)* | 1 | | *5*·*2 (NA)* | 5 |
| M9 | 8 | *6*·*9 (±1*·*1)* | 0 | |  | 8 |
| M12 | 4 | *7*·*6 (±1*·*9)* | 1 | | *7*·*5 (NA)* | 5 |
| M15 | 3 | *7*·*9 (±2*·*1)* | 2 | | *6*·*4 (±1*·*2)* | 5 |
| M18 | 4 | *6*·*9 (±1*·*6)* | 1 | | *5*·*5 (NA)* | 5 |
| Total | 26 |  | 5 | |  | 31 |
| Incidence short disease flares after 18 months in DR: 26/164 = 0·1585 = 15·9% | | | | | | |
| Incidence short disease flares after 18 months in UC: 5/80 = 0·0625 = 6·3% | | | | | | |
| PP1 | | | | | | |
|  | **Number of short disease flares (PASI>5) in DR** | ***Mean (±SD) PASI of the patients with short disease flare in DR*** | **Number of short disease flares (PASI>5) in UC** | | ***Mean (±SD) PASI of the patients with short disease flare in UC*** | **Total** |
| M0 | 0 |  | 0 | |  | 0 |
| M3 | 3 | *11*·*9 (±8*·*7)* | 0 | |  | 3 |
| M6 | 4 | *6*·*4 (±1*·*9)* | 1 | | *5*·*2 (NA)* | 5 |
| M9 | 7 | *6*·*7 (±1*·*1)* | 0 | |  | 7 |
| M12 | 2 | *8*·*3 (±2*·*8)* | 1 | | *7*·*5 (NA)* | 3 |
| M15 | 2 | *8*·*4 (±2*·*7)* | 2 | | *6*·*4 (±1*·*2)* | 4 |
| M18 | 2 | *7*·*6 (±2*·*3)* | 0 | |  | 2 |
| Total | 20 |  | 4 | |  | 24 |
| Incidence short disease flares after 18 months in DR: 20/144 = 0·1389 = 13·9% | | | | | | |
| Incidence short disease flares after 18 months in UC: 4/69 = 0·0579 = 5·8% | | | | | | |
| PP2 | | | | | | |
|  | **Number of short disease flares (PASI>5) in DR** | ***Mean (±SD) PASI of the patients with short disease flare in DR*** | | **Number of short disease flares (PASI>5) in UC** | ***Mean (±SD) PASI of the patients with short disease flare in UC*** | **Total** |
| M0 | 0 |  | | 0 |  | 0 |
| M3 | 3 | *11*·*9 (±8*·*7)* | | 0 |  | 3 |
| M6 | 4 | *6*·*4 (±1*·*9)* | | 1 | *5*·*2 (NA)* | 5 |
| M9 | 7 | *6*·*7 (±1*·*1)* | | 0 |  | 7 |
| M12 | 2 | *8*·*3 (±2*·*8)* | | 1 | *7*·*5 (NA)* | 3 |
| M15 | 2 | *8*·*4 (±2*·*7)* | | 2 | *6*·*3 (±1*·*2)* | 4 |
| M18 | 2 | *7*·*6 (±2*·*3)* | | 1 | *5*·*5 (NA)* | 3 |
| Total | 20 |  | | 5 |  | 25 |
| Incidence short disease flares after 18 months in DR: 20/148 = 0·1351 = 13·5% | | | | | | |
| Incidence short disease flares after 18 months in UC: 5/73 = 0·0685 = 6·9% | | | | | | |

Supplementary Table 4: Proportion of patients with successful dose reduction ***(DR) at month 12 and 18 in the intention-to-treat analysis with last observation carried forward (ITT_LOCF), per protocol 1 analysis (PP1), and per protocol 2 analysis (PP2).***

| ITT_LOCF | | | |
| --- | --- | --- | --- |
|  | ***After 12 months*** | ***After 18 months*** | |
| Total number of patients in DR group | 164 | 164 | |
| Number of patients with DR (step 1 or 2 or lower) | 128 | 123 | |
| Number of patients with successful DR (step 1 or 2 or lower and PASI ≤ 5) | 125 | 120 | |
| Proportion of patients with successful DR | **125/164 = 0·7622 = 76·2%** | **120/164 = 0·7317 = 73·2%** | |
| Number of patients successful on step 1 DR | 34 | 37 | |
| Number of patient successful on step 2 DR | 91 | 83 | |
| Proportion of patients with successful DR per step | **Step 1: 34/164 = 0·2073 = 20·7%**  **Step 2: 91/164 = 0·5549 = 55·5%** | **Step 1: 37/164 = 0·2256 = 22·6%**  **Step 2: 83/164 = 0·5061 = 50·6%** | |
| PP1 | | | |
|  | ***After 12 months*** | | ***After 18 months*** |
| Total number of patients in DR group | 150 | | 144 |
| Number of patients with DR (step 1 or 2 or lower) | 117 | | 108 |
| Number of patients with successful DR (step 1 or 2 or lower and PASI ≤ 5) | 116 | | 107 |
| Proportion of patients with successful DR | **116/150 = 0·7733 = 77·3%** | | **107/144 = 0·7431 = 74·3%** |
| Number of patients successful on step 1 DR | 28 | | 28 |
| Number of patients successful on step 2 DR | 88 | | 79 |
| Proportion of patients with successful DR per step | **Step 1: 28/150 = 0·1867 = 18·7%**  **Step 2: 88/150 = 0·5867 = 58·7%** | | **Step 1: 28/144 = 0·1944 = 19·4%**  **Step 2: 79/144 = 0·5486 = 54·9%** |
| PP2 | | | |
|  | ***After 12 months*** | | ***After 18 months*** |
| Total number of patients in DR group | 151 | | 148 |
| Number of patients with DR (step 1 or 2 or lower) | 117 | | 109 |
| Number of patients with successful DR (step 1 or 2 or lower and PASI ≤ 5) | 116 | | 108 |
| Proportion of patients with successful DR | **116/151 = 0·7682 = 76·8%** | | **108/148 = 0·7297 = 73·0%** |
| Number of patients successful on step 1 DR | 28 | | 29 |
| Number of patients successful on step 2 DR | 88 | | 79 |
| Proportion of patients with successful DR per step | **Step 1: 28/151 = 0·1854 = 18·5%**  **Step 2: 88/151 = 0·5828 = 58·3%** | | **Step 1: 29/148 = 0·1959 = 19·6%**  **Step 2: 79/148 = 0·5338 = 53·4%** |

*Supplementary Table 5: Proportions of patients with successful dose reduction* **(DR) after 12 and 18 months split per biologic, and split per biologic class (all IL-17 inhibitors (IL17i) and all IL-23 inhibitors (IL23i)) in the intention-to-treat analysis with last observation carried forward (ITT_LOCF), per protocol 1 analysis (PP1), and per protocol 2 analysis (PP2).**

| **ITT_LOCF after 12 months** | | | | |
| --- | --- | --- | --- | --- |
| **Biologic** | **DR study completed M12** | **Successful DR step 1 M12** | **Successful DR step 2 M12** | **Proportions of patients per biologic** |
| **IL17i** | | | | |
| Secukinumab | 24 | 5 | 15 | Step 1: 5/24 = **20·8%**  Step 2: 15/24 = **62·5%** |
| Ixekizumab | 41 | 11 | 13 | Step 1: 11/41 = **26·8%**  Step 2: 13/41 = **31·7%** |
| Brodalumab | 10 | 0 | 5 | Step 1: 0/10 = **0·0%**  Step 2: 5/10 = **50·0%** |
| Bimekizumab | 2 | 0 | 2 | Step 1: 0/2 = **0·0%**  Step 2: 2/2 = **100·0%** |
| **Total of all**  **IL17i** | **77** | **16/77 = 0·2078 = 20·8%** | **35/77 = 0·4545 = 45·5%** |  |
|  |  | **(16+35)/77 = 51/77 = 66·2%** | |  |
| **IL23i** | | | | |
| Guselkumab | 48 | 10 | 30 | Step 1: 10/48 = **20·8%**  Step 2: 30/48 = **62·5%** |
| Risankizumab | 35 | 8 | 23 | Step 1: 8/35 = **22·9%**  Step 2: 23/35 = **65·7%** |
| Tildrakizumab | 4 | 0 | 3 | Step 1: 0/4 = **0·0%**  Step 2: 3/4 = **75·0%** |
| **Total of all IL23i** | **87** | **18/87 = 0·2069 = 20·7%** | **56/87 = 0·6437 = 64·4%** |  |
|  |  | **(18+56)/87 = 74/87 = 85·1%** | |  |
| **ITT_LOCF after 18 months** | | | | |
| **Biologic** | **DR study completed M18** | **Successful DR step 1 M18** | **Successful DR step 2 M18** | **Proportions of patients per biologic** |
| **IL17i** | | | | |
| Secukinumab | 24 | 7 | 11 | Step 1: 7/24 = **29·2%**  Step 2: 11/24 = **45·8%** |
| Ixekizumab | 41 | 8 | 15 | Step 1: 8/41 = **19·5%**  Step 2: 15/41 = **36·6%** |
| Brodalumab | 10 | 2 | 2 | Step 1: 2/10 = **20·0%**  Step 2: 2/10 = **20·0%** |
| Bimekizumab | 2 | 1 | 1 | Step 1: 1/2 = **50·0%**  Step 2: 1/2 = **50·0%** |
| **Total of all**  **IL17i** | **77** | **18/77 = 0·2338 = 23·4%** | **29/77 = 0·3766 = 37·7%** |  |
|  |  | **(18+29)/77 = 47/77 = 61·0%** | |  |
| **IL23i** | | | | |
| Guselkumab | 48 | 12 | 29 | Step 1: 12/48 = **25·0%**  Step 2: 29/48 = **60·4%** |
| Risankizumab | 35 | 6 | 23 | Step 1: 6/35 = **17·1%**  Step 2: 23/35 = **65·7%** |
| Tildrakizumab | 4 | 1 | 2 | Step 1: 1/4 = **25·0%**  Step 2: 2/4 = **50·0%** |
| **Total of all IL23i** | **87** | **19/87 = 0·2184 = 21·8%** | **54/87 = 0·6207 = 62·1%** |  |
|  |  | **(19+54)/87 = 73/87 = 83·9%** | |  |

| **PP1 after 12 months** | | | | |
| --- | --- | --- | --- | --- |
| **Biologic** | **DR study completed M12** | **Successful DR step 1 M12** | **Successful DR step 2 M12** | **Proportions of patients per biologic** |
| **IL17i** | | | | |
| Secukinumab | 20 | 3 | 14 | Step 1: 3/20 = **15·0%**  Step 2: 14/20 = **70·0%** |
| Ixekizumab | 39 | 10 | 13 | Step 1: 10/39 = **25·6%**  Step 2: 13/39 = **33·3%** |
| Brodalumab | 9 | 0 | 5 | Step 1: 0/9 = **0·0%**  Step 2: 5/9 = **55·6%** |
| Bimekizumab | 2 | 0 | 2 | Step 1: 0/2 = **0·0%**  Step 2: 2/2 = **100·0%** |
| **Total of all**  **IL17i** | **70** | **13/70 = 0·1857 = 18·6%** | **34/70 = 0·4857 = 48·6%** |  |
|  |  | **(13+34)/70 = 47/70 = 67·1%** | |  |
| **IL23i** | | | | |
| Guselkumab | 44 | 8 | 29 | Step 1: 8/44 = **18·2%**  Step 2: 29/44 = **65·9%** |
| Risankizumab | 32 | 7 | 22 | Step 1: 7/32 = **21·9%**  Step 2: 22/32 = **68·8%** |
| Tildrakizumab | 4 | 0 | 3 | Step 1: 0/4 = **0·0%**  Step 2: 3/4 = **75·0%** |
| **Total of all IL23i** | **80** | **15/80 = 0·1875 = 18·8%** | **54/80 = 0·6750 = 67·5%** |  |
|  |  | **(15+54)/80 = 69/80 = 86·3%** | |  |
| **PP1 after 18 months** | | | | |
| **Biologic** | **DR study completed M18** | **Successful DR step 1 M18** | **Successful DR step 2 M18** | **Proportions of patients per biologic** |
| **IL17i** | | | | |
| Secukinumab | 19 | 5 | 10 | Step 1: 5/19 = **26·3%**  Step 2: 10/19 = **52·6%** |
| Ixekizumab | 37 | 6 | 14 | Step 1: 6/37 = **16·2%**  Step 2: 14/37 = **37·8%** |
| Brodalumab | 9 | 2 | 2 | Step 1: 2/9 = **22·2%**  Step 2: 2/9 = **22·2%** |
| Bimekizumab | 2 | 1 | 1 | Step 1: 1/2 = **50·0%**  Step 2: 1/2 = **50·0%** |
| **Total of all**  **IL17i** | **67** | **14/67 = 0·2090 = 20·9%** | **27/67 = 0·4030 = 40·3%** |  |
|  |  | **(14+27)/67 = 41/67 = 61·2%** | |  |
| **IL23i** | | | | |
| Guselkumab | 42 | 8 | 28 | Step 1: 8/42 = **19·0%**  Step 2: 28/42 = **66·7%** |
| Risankizumab | 31 | 5 | 22 | Step 1: 5/31 = **16·1%**  Step 2: 22/31 = **71·0%** |
| Tildrakizumab | 4 | 1 | 2 | Step 1: 1/4 = **25·0%**  Step 2: 2/4 = **50·0%** |
| **Total of all IL23i** | **77** | **14/77 = 0·1818 = 18·2%** | **52/77 = 0·6753 = 67·5%** |  |
|  |  | **(14+52)/77 = 66/77 = 85·7%** | |  |

| **PP2 after 12 months** | | | | |
| --- | --- | --- | --- | --- |
| **Biologic** | **DR study completed M12** | **Successful DR step 1 M12** | **Successful DR step 2 M12** | **Proportions of patients per biologic** |
| **IL17i** | | | | |
| Secukinumab | 20 | 3 | 14 | Step 1: 3/20 = **15·0%**  Step 2: 14/20 = **70·0%** |
| Ixekizumab | 39 | 10 | 13 | Step 1: 10/39 = **25·6%**  Step 2: 13/39 = **33·3%** |
| Brodalumab | 10 | 0 | 5 | Step 1: 0/10 = **0·0%**  Step 2: 5/10 = **50·0%** |
| Bimekizumab | 2 | 0 | 2 | Step 1: 0/2 = **0·0%**  Step 2: 2/2 = **100·0%** |
| **Total of all**  **IL17i** | **71** | **13/71 = 0·1831 = 18·3%** | **34/71 = 0·4789 = 47·9%** |  |
|  |  | **(13+34)/71 = 47/71 = 66·2%** | |  |
| **IL23i** | | | | |
| Guselkumab | 44 | 8 | 29 | Step 1: 8/44 = **18·2%**  Step 2: 29/44 = **65·9%** |
| Risankizumab | 32 | 7 | 22 | Step 1: 7/32 = **21·9%**  Step 2: 22/32 = **68·8%** |
| Tildrakizumab | 4 | 0 | 3 | Step 1: 0/4 = **0·0%**  Step 2: 3/4 = **75·0%** |
| **Total of all IL23i** | **80** | **15/80 = 0·1875 = 18·8%** | **54/80 = 0·6750 = 67·5%** |  |
|  |  | **(15+54)/80= 69/80 = 86·3%** | |  |
| **PP2 after 18 months** | | | | |
| **Biologic** | **DR study completed M18** | **Successful DR step 1 M18** | **Successful DR step 2 M18** | **Proportions of patients per biologic** |
| **IL17i** | | | | |
| Secukinumab | 20 | 5 | 10 | Step 1: 5/20 = **25·0%**  Step 2: 10/20 = **50·0%** |
| Ixekizumab | 37 | 6 | 14 | Step 1: 6/37 = **16·2%**  Step 2: 14/37 = **37·8%** |
| Brodalumab | 10 | 2 | 2 | Step 1: 2/10 = **20·0%**  Step 2: 2/10 = **20·0%** |
| Bimekizumab | 2 | 1 | 1 | Step 1: 1/2 = **50·0%**  Step 2: 1/2 = **50·0%** |
| **Total of all**  **IL17i** | **69** | **14/69 = 0·2029 = 20·3%** | **27/69 = 0·3913 = 39·1%** |  |
|  |  | **(14+27)/69 = 41/69 = 59·4%** | |  |
| **IL23i** | | | | |
| Guselkumab | 43 | 9 | 28 | Step 1: 9/43 = **20·9%**  Step 2: 28/43 = **65·1%** |
| Risankizumab | 32 | 5 | 22 | Step 1: 5/32 = **14·3%**  Step 2: 22/32 = **68·8%** |
| Tildrakizumab | 4 | 1 | 2 | Step 1: 1/4 = **25·0%**  Step 2: 2/4 = **50·0%** |
| **Total of all IL23i** | **79** | **15/79 = 0·1899 = 19·0%** | **52/79 = 0·6582 = 65·8%** |  |
|  |  | **(15+52)/79 = 67/79 = 84·8%** | |  |

*Supplementary Table 6: Results of the unpaired t-test of the PASI scores* **per visit to test for a significant difference between usual care (UC) and dose reduction (DR) in the intention-to-treat analysis with last observation carried forward (ITT_LOCF), per protocol 1 analysis (PP1), and per protocol 2 analysis (PP2).**

| ITT_LOCF | | | | |
| --- | --- | --- | --- | --- |
| Time point | **PASI Mean (±SD)** | | **t (df)** | **p-value** |
|  | **UC** | **DR** |  |  |
| M0 | 0·7 (±1·0) | 0·6 (±1·0) | 0·9 (242) | p = 0·3840 |
| M3 | 0·8 (±1·0) | 1·0 (±2·1) | -0·8 (242) | p = 0·4280 |
| M6 | 0·8 (±1·1) | 1·0 (±1·5) | -0·9 (207) | p = 0·3870 |
| M9 | 0·7 (±0·9) | 1·1 (±1·7) | -2·2 (240) | **p = 0·0280** |
| M12 | 0·7 (±1·23) | 1·1 (±1·6) | -1·9 (202) | p = 0·0630 |
| M15 | 0·8 (±1·3) | 1·0 (±1·5) | -1·0 (242) | p = 0·3360 |
| M18 | 0·8 (±1·1) | 1·0 (±1·5) | -1·0 (242) | p = 0·3310 |
| PP1 | | | | |
| Time point | **PASI Mean (±SD)** | | **t (df)** | **p-value** |
|  | **UC** | **DR** |  |  |
| M0 | 0·7 (±1·0) | 0·6 (±0·9) | 0·7 (241) | p = 0·4660 |
| M3 | 0·8 (±1·0) | 1·0 (±2·1) | -0·8 (236) | p = 0·4200 |
| M6 | 0·8 (±1·1) | 1·0 (±1·5) | -0·7 (187) | p = 0·4610 |
| M9 | 0·7 (±0·9) | 1·1 (±1·6) | -1·8 (220) | p = 0·0690 |
| M12 | 0·8 (±1·3) | 1·0 (±1·5) | -0·7 (218) | p = 0·4600 |
| M15 | 0·8 (±1·3) | 0·9 (±1·4) | -0·2 (214) | p = 0·8820 |
| M18 | 0·7 (±1·0) | 0·84 (±1·4) | -0·5 (211) | p = 0·6110 |
| PP2 | | | | |
| Time point | **PASI Mean (±SD)** | | **t (df)** | **p-value** |
|  | **UC** | **DR** |  |  |
| M0 | 0·7 (±1·0) | 0·6 (±0·9) | 0·7 (241) | p = 0·4660 |
| M3 | 0·8 (±1·0) | 1·0 (±2·1) | -0·9 (237) | p = 0·3940 |
| M6 | 0·8 (±1·1) | 1·0 (±1·5) | -0·9 (198) | p = 0·3700 |
| M9 | 0·7 (±0·9) | 1·1 (±1·6) | -1·9 (226) | p = 0·0530 |
| M12 | 0·8 (±1·2) | 1·0 (±1·5) | -1·0 (223) | p = 0·3180 |
| M15 | 0·8 (±1·3) | 0·9 (±1·4) | -0·3 (219) | p = 0·7830 |
| M18 | 0·8 (±1·1) | 0·9 (±1·4) | -0·2 (219) | p = 0·8360 |

*Supplementary Table 7: Analysis of covariance (ANCOVA) of PASI and DLQI scores* **between dose reduction (DR) and usual care (UC) at month 12 and month 18 for the per protocol 1 analysis (PP1), and the per protocol 2 analysis (PP2).**

| **PP1** | | | |
| --- | --- | --- | --- |
|  | **DR mean (±SD)** | **UC mean (±SD)** | **p-value** |
| PASI M12 | 1·0 (±1·4) | 0·8 (±1·4) | 0·3000 |
| PASI M18 | 0·9 (±1·3) | 0·7 (±1·3) | 0·4630 |
| DLQI M12 | 1·2 (±2·3) | 0·7 (±2·3) | 0·1680 |
| DLQI M18 | 0·9 (±1·8) | 0·6 (±1·8) | 0·2230 |
| **PP2** | | | |
|  | **DR mean (±SD)** | **UC mean (±SD)** | **p-value** |
| PASI M12 | 1·0 (±1·4) | 0·7 (±1·4) | 0·1960 |
| PASI M18 | 0·9 (±1·3) | 0·8 (±1·4) | 0·6150 |
| DLQI M12 | 1·2 (±2·2) | 0·7 (±2·3) | 0·1430 |
| DLQI M18 | 1·0 (±1·8) | 0·7 (±1·8) | 0·2550 |

*Supplementary Table 8: Safety of dose reduction (DR) versus usual care (UC)* **in the per-protocol 1 analysis (PP1), and per-protocol 2 analysis (PP2).**

| **PP1** | | | |
| --- | --- | --- | --- |
|  | **Event rate per 100 observation years (95%CI)** | | **Rate ratio (95%CI) for DR vs UC** |
|  | **DR group (N=163)** | **UC group (N=75)** |  |
| Total SAE | 4·8 (2·4–8·0) | 3·8 (1·0–8·3) | 1·3 (0·4–4·0) |
| Total AE | 114·1 (100·7–128·4) | 129·0 (108·3–151·5) | 0·9 (0·7–1·1) |
| Total AEoSI | 73·9 (63·2–85·5) | 86·7 (69·9–105·2) | 0·9 (0·7–1·1) |
| *Infection | 48·1 (39·5–57·5) | 63·1 (48·9–79·1) | 0·8 (0·6–1·0) |
| *Malignancy | 1·7 (0·5–3·8) | *NA* | *NA* |
| *Worsening pre-existent PsA | 2·2 (0·7–4·5) | 3·8 (1·0–8·3) | 0·6 (0·2–2·2) |
| *PsA (new diagnosis) | 1·7 (0·5–3·8) | *NA* | *NA* |
| *Musculoskeletal complaints (excluding PsA or trauma) | 15·7 (11·0–21·3) | 15·1 (8·6–23·3) | 1·0 (0·6–1·9) |
| *Hypersensitivity reaction to biologic (including injection site reaction) | 1·3 (0·3–3·2) | 0·9 (0·0–3·5) | 1·4 (0·1–13·4) |
| *Autoimmune disease (excluding PsA) | 0·4 (0·0–1·6) | *NA* | *NA* |
| *Pustular psoriasis | 0·4 (0·0–1·6) | *NA* | *NA* |
| *Psychologic complaints | 0·4 (0·0–1·6) | 0·9 (0·0–3·5) | 0·5 (0·0–7·4) |
| *CVA | 0·9 (0·1–2·4) | 1·9 (0·2–5·2) | 0·5 (0·1–3·3) |
| *Myocardial infarction | 0·9 (0·1–2·4) | 0·9 (0·0–3·5) | 0·9 (0·1–10·2) |
| Neurologic event (without musculoskeletal or CVA) | 2·6 (1·0–5·1) | 1·9 (0·2–5·2) | 1·4 (0·3–7·0) |
| Gastrointestinal (including hepatologic) | 3·5 (1·5–6·3) | 4·7 (1·5–9·6) | 0·7 (0·2–2·3) |
| Gyn-urological | 3·1 (1·2–5·7) | *NA* | *NA* |
| Unexpected reaction to vaccine | 0·4 (0·0–1·6) | *NA* | *NA* |
| Other | 30·6 (23·9–38·2) | 35·8 (25·3–48·0) | 0·9 (0·6–1·3) |
| **PP2** | | | |
|  | **Event rate per 100 observation years (95%CI)** | | **Rate ratio (95%CI) for DR vs UC** |
|  | **DR group (N=163)** | **UC group (N=76)** |  |
| Total SAE | 4·7 (2·4–7·9) | 3·6 (1·0–7·9) | 1·3 (0·4–4·1) |
| Total AE | 113·4 (100·1–127·5) | 126·8 (106·7–148·7) | 0·9 (0·7–1·1) |
| Total AEoSI | 72·9 (62·3–84·3) | 84·2 (68·0–102·2) | 0·9 (0·7–1·1) |
| *Infection | 47·4 (39·0–56·7) | 60·7 (47·0–76·1) | 0·8 (0·6–1·1) |
| *Malignancy | 1·7 (0·5–3·8) | *NA* | *NA* |
| *Worsening pre-existent PsA | 2·2 (0·7–4·4) | 3·6 (1·0–7·9) | 0·6 (0·2–2·2) |
| *PsA (new diagnosis) | 1·7 (0·5–3·8) | *NA* | *NA* |
| *Musculoskeletal complaints (excluding PsA or trauma) | 15·5 (10·9–21·0) | 15·4 (9·0–23·5) | 1·0 (0·6–1·8) |
| *Hypersensitivity reaction to biologic (including injection site reaction) | 1·3 (0·3–3·1) | 0·9 (0·0–3·3) | 1·4 (0·1–13·7) |
| *Autoimmune disease (excluding PsA) | 0·4 (0·0–1·6) | *NA* | *NA* |
| *Pustular psoriasis | 0·4 (0·0–1·6) | *NA* | *NA* |
| *Psychologic complaints | 0·4 (0·0–1·6) | 0·9 (0·0–3·3) | 0·5 (0·0–7·6) |
| *CVA | 0·9 (0·1–2·4) | 1·8 (0·2–5·0) | 0·5 (0·1–3·4) |
| *Myocardial infarction | 0·9 (0·1–2·4) | 0·9 (0·0–3·3) | 1·0 (0·1–10·5) |
| Neurologic event (without musculoskeletal or CVA) | 2·6 (1·0–5·0) | 1·8 (0·2–5·0) | 1·4 (0·3–7·1) |
| Gastrointestinal (including hepatologic) | 3·9 (1·8–6·8) | 4·5 (1·5–9·3) | 0·9 (0·3–2·6) |
| Gyn-urological | 3·0 (1·2–5·6) | *NA* | *NA* |
| Unexpected reaction to vaccine | 0·4 (0·0–1·6) | *NA* | *NA* |
| Other | 30·6 (23·9–38·1) | 36·2 (25·9–48·3) | 0·8 (0·6–1·2) |

Supplementary Table 9: Baseline characteristics of the patients with successful, and unsuccessful DR ***after 18 months in the intention-to-treat analysis with last observation carried forward (LOCF).***

|  | **Successful DR (N=120)** | **Unsuccessful DR (N=44)** |
| --- | --- | --- |
| Age (years) | 49±15 | 50±15 |
| Male | 63.3% (76) | 68.2% (30) |
| BMI | 26.6 [5.6] | 27.6 [5.0] |
| Age at onset of psoriasis | 25±16 | 28±16 |
| Disease duration (years) | 22 [21] | 18 [22] |
| Time of current biologic use (years) | 1.0 [2.0] | 1.0 [2.0] |
| PASI | 0.0 [0.6] | 0.8 [1.8] |
| DLQI | 0.0 [1.0] | 0.0 [1.0] |
| Biologic naivety | 53.3% (64) | 40.9% (18) |
| **Concomitant use of conventional systemic therapy at start of study** | | |
| Methotrexate | 0.8% (1) | 2.3% (1) |
| Acitretin | 0.0% (0) | 0.0% (0) |
| **Number of previously used treatments per patient** | | |
| Conventional systemic | 2.0 [1.0] | 2.0 [1.0] |
| Biologic/SMI | 0.0 [1.0] | 1.0 [2.8] |
| **Comorbidities of interest** | | |
| Cardiovascular disease | 28.3% (34) | 47.7% (21) |
| Depression/anxiety | 18.3% (22) | 15.9% (7) |
| Rheumatologic condition – concomitant PsA | 10.0% (12) | 20.5% (9) |
| Rheumatologic condition – not PsA | 2.5% (3) | 15.9% (7) |
| Diabetes mellitus (type1/2) | 12.5% (15) | 9.1% (4) |
| Inflammatory bowel disease | 0.0% (0) | 2.3% (1) |
| Malignancy | 3.3% (4) | 0.0% (0) |
| Non-melanoma skin cancer | 1.7% (2)* | 0.0% (0) |

Supplementary Figure 1: Overview of the dose reduction strategy and study measures according to the study protocol***.***

Supplementary Figure 2: Sensitivity analysis with additional non-inferiority margins ***below the pre-defined margin of 15·0% (grey line) showing the difference in proportion of persistent flares between dose reduction (DR) and usual care (UC) after 18 months for all analyses.***

PP1_LOCF analysis

PP2_LOCF analysis

ITT_LOCF analysis

ITT_MI analysis

0%

5%

10%

15%

20%

-5%

-10%

-15%

-20%

*Difference in proportion of persistent flares between DR and UC*

Supplementary Figure 3: Kaplan-Meier curve showing time to persistent flare ***for the dose reduction (DR) group and the usual care (UC) group in (a) the intention-to-treat analysis with last observation carried forward (ITT_LOCF), (b) the per protocol 1 analysis (PP1), and (c) per protocol 2 analysis (PP2).***

+

+

0

.

0

0

0

.

2

5

0

.

5

0

0

.

7

5

1

.

0

0

0

3

6

9

1

2

1

5

1

8

T

i

m

e

(

m

o

n

t

h

s

)

S

u

r

v

i

v

a

l

P

r

o

b

a

b

i

l

i

t

y

8

0

8

0

8

0

8

0

8

0

7

9

7

8

1

6

4

1

6

4

1

6

3

1

6

2

1

6

0

1

5

9

1

5

8

D

o

s

e

r

e

d

u

c

t

i

o

n

U

s

u

a

l

c

a

r

e

0

3

6

9

1

2

1

5

1

8

T

i

m

e

(

m

o

n

t

h

s

)

N

u

m

b

e

r

a

t

r

i

s

k

I

T

T

_

L

O

C

F

a

n

a

l

y

s

i

s

+

+

U

s

u

a

l

c

a

r

e

D

o

s

e

r

e

d

u

c

t

i

o

n

a.

(0)

(0)

(0)

(0)

(0)

(0)

(0)

(0)

(0)

(0)

(0)

(0)

(0)

(0)

(cumulative number of censoring)

+

+

+

+

+

+

+

+

+

+

+

+

+

+

+

+

+

+

+

+

0

.

0

0

0

.

2

5

0

.

5

0

0

.

7

5

1

.

0

0

0

3

6

9

1

2

1

5

1

8

T

i

m

e

(

m

o

n

t

h

s

)

S

u

r

v

i

v

a

l

P

r

o

b

a

b

i

l

i

t

y

7

9

7

7

7

3

7

1

7

0

6

9

6

8

1

6

4

1

6

2

1

5

6

1

5

3

1

4

7

1

4

2

1

4

1

D

o

s

e

r

e

d

u

c

t

i

o

n

U

s

u

a

l

c

a

r

e

0

3

6

9

1

2

1

5

1

8

T

i

m

e

(

m

o

n

t

h

s

)

N

u

m

b

e

r

a

t

r

i

s

k

P

P

1

a

n

a

l

y

s

i

s

+

+

U

s

u

a

l

c

a

r

e

D

o

s

e

r

e

d

u

c

t

i

o

n

b.

+

+

+

+

+

+

+

+

+

+

+

+

+

+

+

0

.

0

0

0

.

2

5

0

.

5

0

0

.

7

5

1

.

0

0

0

3

6

9

1

2

1

5

1

8

T

i

m

e

(

m

o

n

t

h

s

)

S

u

r

v

i

v

a

l

P

r

o

b

a

b

i

l

i

t

y

7

9

7

9

7

6

7

5

7

3

7

2

7

1

1

6

4

1

6

2

1

5

6

1

5

3

1

4

8

1

4

5

1

4

5

D

o

s

e

r

e

d

u

c

t

i

o

n

U

s

u

a

l

c

a

r

e

0

3

6

9

1

2

1

5

1

8

T

i

m

e

(

m

o

n

t

h

s

)

N

u

m

b

e

r

a

t

r

i

s

k

P

P

2

a

n

a

l

y

s

i

s

+

+

U

s

u

a

l

c

a

r

e

D

o

s

e

r

e

d

u

c

t

i

o

n

c.

Supplementary Figure 4: Course of mean PASI and DLQI scores ***over 18 months study follow-up in the per protocol 1 analysis (PP1) (a + b), and in the per protocol 2 analysis (PP2) (c + d).***

0

1

2

3

4

5

0

3

6

9

1

2

1

5

1

8

T

i

m

e

(

m

o

n

t

h

s

)

M

e

a

n

P

A

S

I

s

c

o

r

e

R

a

n

d

o

m

i

s

e

d

G

r

o

u

p

U

s

u

a

l

C

a

r

e

D

o

s

e

R

e

d

u

c

t

i

o

n

PP1 analysis

a.

0

1

2

3

4

5

0

3

6

9

1

2

1

5

1

8

T

i

m

e

(

m

o

n

t

h

s

)

M

e

a

n

D

L

Q

I

s

c

o

r

e

R

a

n

d

o

m

i

s

e

d

G

r

o

u

p

U

s

u

a

l

C

a

r

e

D

o

s

e

R

e

d

u

c

t

i

o

n

PP1 analysis

b.

0

1

2

3

4

5

0

3

6

9

1

2

1

5

1

8

T

i

m

e

(

m

o

n

t

h

s

)

M

e

a

n

P

A

S

I

s

c

o

r

e

R

a

n

d

o

m

i

s

e

d

G

r

o

u

p

U

s

u

a

l

C

a

r

e

D

o

s

e

R

e

d

u

c

t

i

o

n

PP2 analysis

c.

0

1

2

3

4

5

0

3

6

9

1

2

1

5

1

8

T

i

m

e

(

m

o

n

t

h

s

)

M

e

a

n

D

L

Q

I

s

c

o

r

e

R

a

n

d

o

m

i

s

e

d

G

r

o

u

p

U

s

u

a

l

C

a

r

e

D

o

s

e

R

e

d

u

c

t

i

o

n

PP2 analysis

d.

Supplementary Figure 5: Course of PASI scores on individual patient level ***over 18 months study follow-up in the intention-to-treat analysis with last observation carried forward (ITT_LOCF) (a), per protocol 1 analysis (PP1) (b), and in the per protocol 2 analysis (PP2) (c).***

0

1

2

3

4

5

0

3

6

9

1

2

1

5

1

8

T

i

m

e

(

m

o

n

t

h

s

)

P

A

S

I

s

c

o

r

e

R

a

n

d

o

m

i

s

e

d

G

r

o

u

p

U

s

u

a

l

C

a

r

e

D

o

s

e

R

e

d

u

c

t

i

o

n

ITT_LOCF analysis

a.

22

10

0

1

2

3

4

5

0

3

6

9

1

2

1

5

1

8

T

i

m

e

(

m

o

n

t

h

s

)

P

A

S

I

s

c

o

r

e

R

a

n

d

o

m

i

s

e

d

G

r

o

u

p

U

s

u

a

l

C

a

r

e

D

o

s

e

R

e

d

u

c

t

i

o

n

PP1 analysis

b.

22

10

0

1

2

3

4

5

0

3

6

9

1

2

1

5

1

8

T

i

m

e

(

m

o

n

t

h

s

)

P

A

S

I

s

c

o

r

e

R

a

n

d

o

m

i

s

e

d

G

r

o

u

p

U

s

u

a

l

C

a

r

e

D

o

s

e

R

e

d

u

c

t

i

o

n

PP2 analysis

c.

10

22

Supplementary Figure 6: Kaplan-Meier curve showing time to first dose reescalation ***for the dose reduction (DR) group and the usual care (UC) group in the intention-to-treat analysis with last observation carried forward (ITT_LOCF).***

+

0

.

0

0

0

.

2

5

0

.

5

0

0

.

7

5

1

.

0

0

0

3

6

9

1

2

1

5

1

8

T

i

m

e

(

m

o

n

t

h

s

)

1

6

4

1

6

4

1

5

9

1

4

4

1

2

2

1

0

7

91

D

o

s

e

r

e

d

u

c

t

i

o

n

0

3

6

9

1

2

1

5

1

8

T

i

m

e

(

m

o

n

t

h

s

)

N

u

m

b

e

r

a

t

r

i

s

k

I

T

T

_

L

O

C

F

a

n

a

l

y

s

i

s

+

D

o

s

e

r

e

d

u

c

t

i

o

n

Survival probability

(0)

(0)

(0)

(0)

(0)

(0)

(0)
