# Supplementary material for: Dose Reduction of IL17 and IL23 Inhibitors in Psoriasis (BeNeBio study): An International, Pragmatic, Multicentre, Randomised, Controlled, Non-Inferiority Trial
Source: Lancet Reg Health Eur. 2026 Jun 1;67:101721. doi: 10.1016/j.lanepe.2026.101721 (PMC13453948; doi:10.1016/j.lanepe.2026.101721)
Supplement: Supplementary file 3 [file mmc2.docx]

**Supplementary Appendix 1**

**Statistical Analysis Plan (Protocol-guided reconstruction)**

*The references in this appendix correspond with the reference list in the main text.*

Table of contents

[1. Title and administrative information	2](#_Toc227320762)

[2. Background and purpose	2](#_Toc227320763)

[3. Study design overview	2](#_Toc227320764)

[4. Estimands framework and intercurrent events	2](#_Toc227320765)

[4.1 Primary estimand	2](#_Toc227320766)

[4.2 Intercurrent events	2](#_Toc227320767)

[4.3 Handling of intercurrent events	3](#_Toc227320768)

[5. Analysis populations	3](#_Toc227320769)

[5.1 Intention-to-treat (ITT) population	3](#_Toc227320770)

[5.2 Per-protocol population 1 (PP1)	3](#_Toc227320771)

[5.3 Per-protocol population 2 (PP2)	3](#_Toc227320772)

[5.4 Safety population	3](#_Toc227320773)

[6. Endpoints	3](#_Toc227320774)

[6.1 Primary endpoint	3](#_Toc227320775)

[6.2 Secondary endpoints	3](#_Toc227320776)

[7. Statistical methods	4](#_Toc227320777)

[7.1 General principles	4](#_Toc227320778)

[7.2 Primary outcome analysis	4](#_Toc227320779)

[7.3 Sensitivity analyses for primary outcome	4](#_Toc227320780)

[7.3.1 Unadjusted analysis	4](#_Toc227320781)

[7.3.2 Alternative confidence interval methods	4](#_Toc227320782)

[7.3.3 Alternative non-inferiority margins (exploratory)	4](#_Toc227320783)

[7.4 Time-to-event analysis	4](#_Toc227320784)

[7.5 Secondary outcomes	4](#_Toc227320785)

[7.5.1 Continuous outcomes (PASI, DLQI)	4](#_Toc227320786)

[7.5.2 Binary outcomes	5](#_Toc227320787)

[7.6 Safety analysis	5](#_Toc227320788)

[7.7 Missing data handling	5](#_Toc227320789)

[7.8 Subgroup analyses (exploratory)	5](#_Toc227320790)

[8. Multiplicity	6](#_Toc227320791)

[9. Software and reproducibility	6](#_Toc227320792)

[10. Deviations from original protocol	6](#_Toc227320793)

[11. Limitations of SAP reconstruction	6](#_Toc227320794)

[12. Extended methods section	6](#_Toc227320795)

# **1. Title and administrative information**

This document describes the statistical analysis plan (SAP) for the randomised controlled trial investigating dose reduction (DR) of interleukin-17 inhibitors (IL17i) and interleukin-23 inhibitors (IL23i) compared with usual care (UC) in adult patients with plaque psoriasis and stable low disease activity.

The SAP was not finalised prior to database lock. However, analyses were guided by the originally approved study protocol (2018) and updated in accordance with current methodological standards for non-inferiority trials and estimand-based reporting. All deviations from the original protocol are explicitly documented.

Trial registration: ClinicalTrials.gov NCT04340076

Study period: June 30, 2020 – January 30, 2025

Statistical software: R version 4.5.2; SPSS version 29.0

# **2. Background and purpose**

The purpose of this SAP is to provide a transparent and structured description of all statistical analyses performed in the trial, including:

1. Reconstruction of prespecified analyses from the original protocol
2. Specification of the primary estimand and analysis populations
3. Clear definition of statistical models and methods
4. Documentation of sensitivity analyses
5. Explicit reporting of deviations from the original protocol

This SAP aims to improve transparency and reproducibility without implying that all details were prospectively finalised.

# **3. Study design overview**

This was a multicentre, pragmatic, open-label, randomised controlled non-inferiority trial conducted in 11 Dutch and 8 Belgian hospitals.

- Population: adults with plaque psoriasis treated with IL17i or IL23i
- Randomisation: 2:1 (DR:UC), block randomisation with variable block sizes (6, 9, 12)
- Stratification: biologic
- Follow-up: 18 months
- Masking: none (open-label pragmatic design)

# **4. Estimands framework and intercurrent events**

## **4.1 Primary estimand**

The primary estimand reflects a treatment-policy strategy, estimating the effect of initiating DR versus UC on the incidence of persistent flares over 18 months, regardless of treatment modifications (i.e. intercurrent events) after randomisation in all randomised patients,

## **4.2 Intercurrent events**

Intercurrent events included:

- Continuing DR while PASI>5 and/or DLQI>10
- New DR attempt after returning to the standard or (previous) effective dose (reescalation of the dose)
- Reduce dose while randomised to UC
- Dropout (lost to follow-up)
- Switch of biologic
- Withdrawal from/stop of biologic
- Systematically longer/shorter injection interval than protocol without medical reason

## **4.3 Handling of intercurrent events**

The handling of intercurrent events depended on the analysis population (see Section 5) and is summarised in Supplementary Table 1.

# **5. Analysis populations**

## **5.1 Intention-to-treat (ITT) population**

All randomised patients analysed according to assigned treatment group, regardless of occurrence of intercurrent events (see 4.2 and Supl. Table 1).

## **5.2 Per-protocol population 1 (PP1)**

Strict adherence definition (see 4.2 and Supl. Table 1):

- DR group: censored/excluded after first intercurrent event
- UC group: censored/excluded after any deviation from standard dose of included biologic

## **5.3 Per-protocol population 2 (PP2)**

Pragmatic adherence definition (see 4.2 and Supl. Table 1):

- DR group: censored/excluded after first intercurrent event, except for the following intercurrent events:
  - reattempt of DR after reescalation of the dose in DR group
  - switching of biologic if the DR protocol was followed prior to switching in DR group
- UC group: censored/excluded after lost to follow up, but other treatment modifications are allowed including different dosages.

## **5.4 Safety population**

Safety analyses were done for ITT population, PP1 population and PP2 population. All patients receiving at least one dose of study treatment; but only patients with at least one dose of the study drug were included for each analysis.

# **6. Endpoints**

## **6.1 Primary endpoint**

Incidence proportion of persistent flares at 18 months, defined as:

- PASI >5 for ≥3 consecutive months

## **6.2 Secondary endpoints**

Clinical disease activity

- PASI and DLQI over time
- Time until first persistent flare (PASI >5 for ≥3 consecutive months
- Short disease flares (PASI >5 at a single time point)

Treatment outcomes

- Proportion of successful dose reduction (PASI ≤5 while on reduced dose)
- Initiation of other anti-psoriatic medication

Safety outcomes

- Adverse events (AE)
- Serious adverse events (SAEs); all SAEs and SAEs related to DR
- Adverse events of special interest (AEoSI); all AEoSI and AEoSI related to DR

Additional outcomes (reported separately; not in current manuscript)

- Pharmacokinetics/pharmacodynamics
- Cost-effectiveness analyses
- Predictors of successful DR

# **7. Statistical methods**

## **7.1 General principles**

- One-sided α = 0·025 for non-inferiority testing
- Two-sided 95% confidence intervals (CI) used for all estimates
- Primary analysis conducted under intention-to-treat principle
- Non-inferiority margin: 15·0%

## **7.2 Primary outcome analysis**

The primary outcome was analysed using marginal standardization based on a generalised linear model for a binary outcome with identity link.

Model specification:

- Outcome: persistent flare (yes/no)
- Predictors:
  - treatment group (DR vs UC)
  - stratification factor (biologic)

Estimation:

- Marginal risks estimated via standardization over empirical covariate distribution
- Risk differences calculated as DR − UC
- 95% CI estimated using delta method

Non-inferiority criterion:

Non-inferiority was concluded if the upper bound of the 95% CI for the risk difference did not exceed 15·0%.

## **7.3 Sensitivity analyses for primary outcome**

### **7.3.1 Unadjusted analysis**

- Crude incidence proportions
- Risk differences without covariate adjustment

### **7.3.2 Alternative confidence interval methods**

- Wilson–Newcombe method (preferred for low event rates)
- Fisher exact / Clopper–Pearson (protocol-consistent legacy approach)

### **7.3.3 Alternative non-inferiority margins (exploratory)**

- 10·0% margin (underpowered, exploratory only)
- 5·0% margin (underpowered, exploratory only)

## **7.4 Time-to-event analysis**

Time to first persistent flare was analysed using Kaplan–Meier survival curves. Group differences were visually assessed over 18 months.

## **7.5 Secondary outcomes**

### **7.5.1 Continuous outcomes (PASI, DLQI)**

- Analysed at 12 and 18 months using ANCOVA
- Adjusted for baseline variables:
  - gender
  - body mass index
  - disease duration
  - concomitant psoriatic arthritis
  - biologic naivety

Between-group comparisons at three-month intervals were assessed using unpaired t-tests.

### **7.5.2 Binary outcomes**

- Initiation of additional systemic therapy
- Analysed descriptively (no formal hypothesis testing)

## **7.6 Safety analysis**

Adverse events were summarised as:

- Incidence proportions
- Rate ratios (RR) with 95% CI using Wald method

SAEs and AEoSI were analysed without imputation.

AEoSI included:

- Infection
- Malignancy
- Worsening of existent psoriatic arthritis
- New onset of psoriatic arthritis
- Musculoskeletal complaints
- Hypersensitivity reaction
- Autoimmune disease
- Pustular psoriasis
- Psychologic complaints
- Cerebrovascular events
- Myocardial infarction

**7.7 Missing data handling**

Intention-to-treat population:

- Primary analysis (first presented version): multiple imputation (MI)
- Supplementary: last observation carried forward (LOCF)

Per-protocol populations:

- LOCF applied until intercurrent event
- No imputation beyond deviation point

Missingness was assumed to be missing at random (MAR) for MI procedures.

## **7.8 Subgroup analyses (exploratory)**

- Biologic class (IL17i vs IL23i) (descriptive only)
- Individual drug analysis (descriptive only)

No formal adjustment for multiplicity was applied.

# **8. Multiplicity**

The primary outcome was the only confirmatory endpoint. All secondary analyses were exploratory and hypothesis-generating.

# **9. Software and reproducibility**

Analyses were performed using R version 4.5.2 and SPSS version 29.0

No independent data monitoring committee was installed; monitoring was conducted according to local regulatory standards.

# **10. Deviations from original protocol**

| Element | Protocol (2018) | Final SAP approach | Rationale |
| --- | --- | --- | --- |
| Primary analysis population | Order of presentation:  1. PP  2. ITT | Order of presentation:  1. ITT  2. PP | Alignment with EMA guidance to put ITT first ^15, 16^ |
| Confidence interval method | Fisher exact / Clopper-Pearson | Wilson–Newcombe + delta method | Improved performance for rare events |
| Missing data | LOCF only | LOCF + multiple imputation | Reduced bias and uncertainty handling^18^ |
| PP analysis structure | Single PP | PP1 + PP2 | More detailed analysis to test different levels of adherence to the protocol (i.e. strict adherence (PP1), vs. less strict adherence (PP2) |
| Adjustment for stratification | Not specified | Included in model | Standard methodological best practice |
| Safety analyses | Differences (proportions) between groups will be tested with Fisher exact test. | Rate ratios (RRs) were compared using Wald test. | A comparison based on proportions of adverse events is less preferable as time at risk varied between individual patients and due to repeating events. Therefore, RRs were compared using the Wald test. |

# **11. Limitations of SAP reconstruction**

This SAP was not finalised prior to database lock. However, analyses were:

- Anchored in the original protocol
- Aligned with current regulatory and methodological guidance
- Supplemented by sensitivity analyses to assess robustness

The main limitation is the absence of full prospective SAP locking; however, transparency in deviations and multiple analytic frameworks mitigates this risk.

**12. Extended methods section**

**Introduction**

This study was designed to investigate if DR of IL17i and IL23i by a disease activity-guided strategy is non-inferior to usual care (UC) regarding persistent disease flares in patients with psoriasis with low disease activity. The initial protocol was designed in 2018, therefore, the final analyses were adapted to be in accordance with current guidelines and current statistical best practice. A standalone statistical analysis plan was not finalised prior to database lock, however, analyses were guided by the previously published study protocol and were updated when deemed necessary.^11^ A detailed and fully transparent description of all statistical methods is provided below, including clarification of all adaptations to the initial protocol.

**Study design**

We conducted a, multicentre, pragmatic, open-label, randomised, controlled, non-inferiority trial of IL17 inhibitors (IL17i) (secukinumab, ixekizumab, brodalumab, bimekizumab) and IL23 inhibitors (IL23i) (guselkumab, risankizumab, tildrakizumab) from June 30, 2020, to January 30, 2025, in 11 Dutch and 8 Belgian (non-)academic hospitals. Patient representatives were involved in all phases of the study. An extended description of the study design, including a rationale for the sample size calculation and non-inferiority margin, is published previously elsewhere (online study protocol).^11^

The study protocol and amendments were approved by the Medical Ethical Committee (Arnhem-Nijmegen, NL71920.091.19) for the Dutch sites and from the competent authorities (FAHMP) and the Ethics Committee of Ghent University Hospital and Ghent University after consulting the Ethics Committees of each Belgian participating site. Study performance was in accordance with Good Clinical Practice guidelines, and the Declaration of Helsinki.^12^ Reporting of this trial is according to the Consolidated Standards of Reporting Trials (CONSORT) reporting guideline. The study is closed and registered in ClinicalTrials.gov (Identifier: NCT04340076; status: closed).

**Patients**

Adult patients with plaque psoriasis using standard, registered doses of IL17i or IL23i for at least 6 months with stable low disease activity (defined as Psoriasis Area and Severity Index (PASI) ≤5 for ≥6 months at inclusion, and Dermatology Life Quality Index (DLQI) ≤5 at inclusion) were included. At regular visits, patients were first asked by their treating physicians if they agreed to be informed on the study. If so, patients were further counselled and eventually recruited by trained research staff (research nurses and physicians), who were also actively involved during study follow-up visits. Patients were excluded if the biologic was primarily prescribed for another disease than plaque psoriasis (e.g. for psoriatic arthritis), if they used concomitant systemic immunosuppressants/therapies (except methotrexate and acitretin when used for psoriasis), or if they had a severe comorbidity with short life expectancy. Sex/gender data were derived from patient records; ethnicity/race data were not collected. All patients provided oral and written informed consent.

**Randomisation and masking**

A total of 244 patients was randomised 2:1 by a web-based program (www.castoredc.com) to DR (N=164) or UC (N=80) by block randomisation (variable block size of 6, 9, 12) with a random allocation sequence, and stratified by biologic. During the entire study, no masking was applied owing to the pragmatic study design aiming for the highest possible external validity. In this way, we aimed to provide a more realistic reflection of how our DR intervention would function in real-world practice rather than under ideal circumstances. Trained physicians or nurses enrolled patients and ensured randomisation by the web-based program, and to ensure internal consistency, they executed the patients’ study follow-up visits.

**Procedures**

The DR strategy and study measures are visualised in Supplementary Figure 1 (SupFig.1). Patients in the UC group received the standard dose of their IL17i or IL23i. Due to the pragmatic design of this study, treatment alterations were allowed when needed as UC represented daily practice care as described previously.^11^ In the DR group, DR was performed by stepwise injection interval prolongation to first 67·0% of the standard dose (1·5-fold extension of the interval in standard dose) (SupFig.1). Disease activity was monitored at every study visit using PASI and DLQI scores, assessed by trained physicians or nurses. The dose was further reduced to 50·0% of the standard dose (2-fold extension of the interval in standard dose) when disease activity remained low (PASI≤5 and DLQI≤5) (SupFig.1). When PASI>5 and/or DLQI>10, or by patient preference, the (previous) effective dose was reinstalled, meaning reescalation of the dose. When PASI≤5 and DLQI>5 but ≤10, it was discussed with the patient if the lowered dose could be continued. After DR failure, and consequently, reescalation, no new DR attempts were advised by the study team. Study visits were three-monthly during a total study follow-up of 18 months for both groups. Additional study visits took place when needed, patients were informed on the possibility to contact the study team in-between visits prior to inclusion. At every study visit, questionnaires were completed, and serum samples collected. In both groups, it was permitted to initiate, continue, or discontinue methotrexate, acitretin, or topical therapies for psoriasis when deemed necessary by the physician.

**Outcomes**

The primary outcome was to assess non-inferiority of DR by the difference in incidence proportion of persistent flares (PASI>5 for ≥3 consecutive months) between the DR and UC group after 18 months. This difference was compared with the non-inferiority margin of 15·0%. If the upper limit of the 95% confidence interval (CI) of the between-group difference exceeded 15·0%, then non-inferiority of DR was not demonstrated. The primary estimand reflects a treatment-policy strategy, estimating the effect of initiating the DR strategy versus UC on the incidence of persistent flares regardless of treatment modifications after randomisation in all randomised patients over 18 months.

Secondary outcomes were:

- proportion of patients with successful DR (using a lower dose and maintaining PASI≤5) after 12 and 18 months,
- between-group difference in PASI scores at each three-monthly time point, and in the course of PASI and DLQI scores over 18 months,
- to assess the time until first persistent flare (PASI >5 for ≥3 consecutive months),
- between-group difference in the incidence of short disease flares (PASI>5 at one time point) after 18 months,
- between-group difference in the proportion of patients with initiation of other anti-psoriatic medication (methotrexate, acitretin, cyclosporin, intensive topical therapies like dithranol),
- serious adverse events (SAE) related to DR and between-group differences in SAE, adverse events (AE), and adverse events of special interest (AEoSI),

Secondary outcomes regarding pharmacokinetic/-dynamic profiles, cost-effectiveness, and predictors of successful DR were also formulated in the study protocol and will be addressed in consecutive manuscripts.

**Statistical Analysis**

The non-inferiority margin of 15·0%, for the difference in the incidence proportion in persistent flares, was based on previous literature^1, 13^, and clinical grounds. Extensive rationale can be found elsewhere.^11^ Based on this margin, one-sided testing (α=0·025; 1–β=0·8), and a 2:1 randomisation ratio of DR to UC, a total of 222 patients were required to reject the null hypothesis of inferiority. Accounting for the anticipated dropout rate, the total sample size increased to 244 patients.

Baseline and treatment characteristics were summarised using descriptive statistics expressed as frequencies (%, (n)), mean with standard deviation (±SD), and median with interquartile range [IQR]. The primary outcome—incidence proportion of persistent flares at 18 months—was summarised for each treatment group as marginal risks (proportions). Treatment effects were expressed as risk differences. Marginal risks and risk differences were estimated using marginal standardization based on a generalised linear model for the binary outcome with an identity contrast. The model included treatment group and stratification factor (biologic), and estimates were standardised over the empirical covariate distribution of the study population. Risk differences and corresponding 95%CI were estimated using the delta method, with variance estimation accounting for the stratification factor. Marginal risks for each treatment group were obtained from the same outcome model by assigning the treatment indicator to each level sequentially, and averaging predicted probabilities across all participants. Correction for stratification factor was not explicitly mentioned in the pre-published study protocol but is a general rule and therefore applied for the primary outcome.^14^ In order to comply with the initial protocol, where the difference in proportions and their corresponding 95%CI would be calculated by Fisher exact tests (Clopper-Pearson), unadjusted risk differences and corresponding 95%CI were also shown, as a sensitivity analysis of the primary outcome. After calculating the unadjusted proportions of persistent flares in both treatment groups, it was decided to use the Wilson-Newcombe method to calculate the risk differences and corresponding 95%CI instead of the Fisher exact tests as this method would be more robust and accurate regarding the low proportions of persistent flares in both treatment groups. In addition to the 15·0% non-inferiority margin, the analyses of the primary outcome were also performed for 10·0% and 5·0% margins as post-hoc sensitivity analyses, however, the study was not powered for these margins. In addition, the 5·0% margin would require unfeasibly large sample sizes, and was found too conservative and not commonly acceptable like the 10·0% and 15·0% margins. Time to the first persistent flare was visualised for both treatment groups using a Kaplan-Meier survival estimation. An intention-to-treat (ITT) analysis was performed as primary analysis. In the initial protocol designed in 2018, the per-protocol (PP) analysis was set as primary method as it was regarded as most conservative. However, according to current EMA guidelines on non-inferiority studies, and evolving insights on estimands, an ITT analysis is preferred as primary analysis as a PP approach could be vulnerable to bias, and is presented as such.^15, 16^ Under specific circumstances, the ITT approach might still attenuate results towards non-inferiority, therefore, two PP analyses (named PP1 and PP2 that differ in population, see below) were also presented.^17^ In light of the primary estimand, the decision whether a patient remained in the analysis was based on intercurrent events, reflecting more than merely strict protocol deviations. Supplementary Table 1 shows a detailed overview of intercurrent events (i.e. the events after randomisation, including lost to follow-up and protocol deviations) and how they were handled per analysis (ITT, PP1, PP2). In the ITT analysis, patients were analysed according to treatment allocation and intercurrent events were accepted as part of real-world practice, which is similar to the initial protocol. In the initial protocol, only one PP analysis was described, however, in light of the pragmatic design of the study, it was decided to perform two PP analyses of which one is strictly according to the initial protocol (PP1), and one would reflect a broader interpretation of the initial protocol (PP2). In the PP1 analysis, patients in the DR group were excluded from analysis from the point of an intercurrent event onward, and patients in the UC group were excluded after any changes to their standard dose (see Supplementary Table 1). As the PP2 analysis reflects a broader interpretation of the protocol which is more in line with the pragmatic design of the study; patients with DR were allowed to make a new DR attempt after reescalation, and to switch to another biologic if the DR protocol was followed prior to switching, and patients in the UC group were allowed to modify their treatment as part of usual care (see Supplementary Table 1). Although patients were excluded from the PP analyses from the point of an intercurrent event onward, they did complete all study procedures. Occurrence of intercurrent events was summarised and reported. Regarding missing data, in the ITT analysis, multiple imputation (ITT_MI) was performed for the primary outcome, and last observation carried forward on all missing time points (ITT_LOCF) for both primary and secondary outcomes. In both the PP analyses, LOCF was performed for both primary and secondary outcomes for missing data until occurrence of an intercurrent event (Supplementary Table 1). The extent and nature of missing data were reported. Although our initial protocol specified LOCF for imputing missing data, we have opted to also include MI to better account for uncertainty, and reduce bias that LOCF may introduce.^18^

In the DR group, the proportion of patients with successful DR after 12 and 18 months was reported descriptively for the total population, for subgroups by drug class, and individual drugs. The between-group difference in PASI scores at each three-monthly time point was analysed by an unpaired t-test. Analysis of covariance (ANCOVA) was performed to test between-group differences in PASI and DLQI scores at 12 and 18 months, adjusted for baseline variables (gender, body mass index, disease duration, concomitant psoriatic arthritis (PsA), and biologic naivety). The difference in the proportion of patients with initiation of other anti-psoriatic medications between DR and UC was analysed descriptively.

Serious adverse events (SAEs), adverse events (AE), and adverse events of special interest (AEoSI) were summarised as proportions (without imputing data for missing time points), and rate ratios (RR) with 95%CI, calculated using the Wald method. According to the initial protocol, differences between treatment groups would be tested using Fisher exact test, however, a comparison based on proportions of adverse events is less preferable as time at risk varied between individual patients and due to repeating events, also, number of SAEs, AE, and AEoSI were sufficiently large and approximately balanced between treatment groups. Therefore, exact methods were not used. AEoSI included infection, malignancy, worsening of existent PsA, new-onset PsA, musculoskeletal complaints, hypersensitivity reaction, autoimmune disease, pustular psoriasis, psychologic complaints, CVA, and myocardial infarction. Additionally, SAEs deemed specifically related to DR itself, as assessed by two reviewers (CR and JR), were analysed.

Statistical analyses were performed with SPSS software, version 29.0 (SPSS Inc), and R, version 4.5.2. No data monitoring committee was installed; the data was monitored by external data-monitors according to local guidelines.
