# Supplementary material for: Dose Reduction of IL17 and IL23 Inhibitors in Psoriasis (BeNeBio study): An International, Pragmatic, Multicentre, Randomised, Controlled, Non-Inferiority Trial
Source: Lancet Reg Health Eur. 2026 Jun 1;67:101721. doi: 10.1016/j.lanepe.2026.101721 (PMC13453948; doi:10.1016/j.lanepe.2026.101721)
Supplement: Supplementary file 4 [file mmc4.docx]

**Supplementary Table 1**

ITT: intention-to-treat, PP: per protocol, DR: dose reduction, UC: usual care, PASI: Psoriasis Area and Severity Index, DLQI: Dermatology Life Quality Index, NA: not applicable, M: visit month.

**Supplementary Table 2:**

PASI: Psoriasis Area and Severity Index, SD: standard deviation, M: visit month, Persistent flare: PASI>5 for ≥3 months. *Short disease flare after previously being returned to the previous effective dose (here 67·0% of standard dose). **PASI<5 not re-established as patient stopped treatment on his own initiative.

**Supplementary Table 3:**

PASI: Psoriasis Area and Severity Index, SD: standard deviation, M: visit month, NA: not applicable, Short disease flare: PASI>5 at one timepoint.

**Supplementary Table 4:**

Successful DR is defined as using 67% of the standard dose (step 1), or 50% (step 2) of the standard dose, and having PASI≤5. N: number of patients, M: visit month, DR: dose reduction, PASI: Psoriasis Activity and Severity Index.

**Supplementary Table 5:**

M: visit month, IL: interleukin.

**Supplementary Table 6:**

PASI: Psoriasis Area and Severity Index, ±SD: standard deviation, df: degree of freedom, M: visit month.

**Supplementary Table 7:**

PASI: Psoriasis Area and Severity Index, DLQI: Dermatology Life Quality Index, ±SD: standard deviation, M: visit month.

**Supplementary Table 8:**

Safety is shown as event rate per 100 observation years and the rate ratio with 95% confidence intervals (CI). SAE: serious adverse events, AE: adverse events, AEoSI: adverse events of special interest, *category included as AEoSI. PsA: psoriatic arthritis, CVA: cerebrovascular event, NA: not applicable.

**Supplementary Table 9:**

Means with standard deviations (±SD), number of patients (n) with percentages (%), and medians with interquartile ranges [IQR]. *both cases concerned basal cell carcinoma. BMI = Body Mass Index, PASI: Psoriasis Area and Severity Index, DLQI: Dermatology Life Quality Index, SMI: Small Molecule Inhibitor, PsA: psoriatic arthritis.

**Supplementary Figure 1:**

R = randomisation, DR = dose reduction, N = number of patients, PASI = Psoriasis Area and Severity Index, DLQI = Dermatology Life Quality Index, M = month, EQ-5D-5L = European Quality of Life-5 Dimensions-5 Level, SF-36 = Short Form 36, iMTA = institute for Medical Technology Assessment, MCQ = Medical Consumption Questionnaire, PCQ = Productivity Cost Questionnaire.

**Supplementary Figure 2:**

Error bars indicate 95% confidence intervals. The orange lines at 10·0% and 5·0% show the non-inferiority margins; confidence intervals crossing the orange lines are not non-inferior. Note that the current sample size is not fit for these smaller non-inferiority margins. In addition, this margin was deemed too conservative regarding clinical relevance in the context of this study. ITT: intention-to-treat, PP: per-protocol, MI: multiple imputation, LOCF: last observation carried forward.

**Supplementary Figure 3:**

Persistent flare: PASI>5 for ≥3 months.

**Supplementary Figure 4:**

The mean PASI and DLQI scores with 95% confidence interval, for both DR and UC group, are shown per visit. DR: dose reduction, UC: usual care, PASI: Psoriasis Area and Severity Index, DLQI: Dermatology Life Quality Index.

**Supplementary Figure 5:**

DR: dose reduction, UC: usual care, PASI: Psoriasis Area and Severity Index, DLQI: Dermatology Life Quality Index.

**Supplementary Figure 6:**

Of note, patients could either reescalate to their previous effective dose or to their original dose (standard dose). They were considered as 'unsuccessful DR' only when they returned to the standard dose, but were considered as ‘successful DR’ when they reescalated only to their previous effective dose (e.g. from 50% to 67% of the original dose).
